# Combined SMRT and Illumina RNA Sequencing Reveals the Alternative Splicing-Mediated Regulation of Anthocyanin Accumulation in Potato (*Solanum tuberosum* L.)

**DOI:** 10.3390/plants15030514

**Published:** 2026-02-06

**Authors:** Minmin Bao, Zhitao Li, Jinyong Zhu, Xiaoqiang Qiu, Yuanming Li, Zhenzhen Bi, Panfeng Yao, Zhen Liu, Yuhui Liu

**Affiliations:** 1 Agronomy College, Gansu Agricultural University, Lanzhou 730070, China; 2 State Key Laboratory of Aridland Crop Science, Gansu Agricultural University, Lanzhou 730070, China; 3 Seed Industry Research Institute of Gansu Provincial University, Gansu Agricultural University, Lanzhou 730070, China; 4 College of Horticulture, Gansu Agricultural University, Lanzhou 730070, China

**Keywords:** potato (*Solanum tuberosum* L.), single-molecule real-time (SMRT) sequencing, alternative splicing, anthocyanin biosynthesis, *StB3-like* transcription factor

## Abstract

Anthocyanins, as natural pigments with high nutritional value, have been extensively studied in terms of their biosynthetic pathways. However, the specific impact of alternative splicing (AS) on anthocyanin biosynthesis in potatoes and its potential regulatory mechanisms remain unclear. This study analyzed full-length transcriptome (SMRT) and Illumina RNA-seq datasets from three developmental stages of multiple potato (*Solanum tuberosum* L.) cultivars. After RNA-seq correction, numerous AS events were identified in both white and colored samples. To dissect the regulatory role of AS in anthocyanin biosynthesis, we further analyzed the AS patterns of anthocyanin biosynthesis-related structural genes and transcription factors (TFs). Through this targeted analysis, we found that a subset of these structural genes and TFs exhibited AS, generating functionally diverse transcript variants. Among these, the candidate gene *StB3-like* (*Soltu.DM.04G010530*), a B3 family TF associated with anthocyanin synthesis, was selected for preliminary functional validation. This gene produced three alternatively spliced transcripts (*StB3-like-1*, *-2*, *-3*). Transient co-expression with *StAN1 (Soltu.DM.10G020850)* in tobacco showed that *StB3-like-2* and *StB3-like-3* significantly increased anthocyanin accumulation, whereas *StB3-like-1* had no effect. These results confirm that different transcripts produced by the alternative splicing of the same gene differentially regulate anthocyanin accumulation in a *StAN1*-dependent manner, laying the groundwork for further investigation into the role of alternative splicing in potato anthocyanin accumulation.

## 1. Introduction

Originating from the Andean regions of Chile and Peru, potato (*Solanum tuberosum* L.) is an annual tuber crop of the Solanaceae family. It is widely cultivated in over 150 countries and regions globally, owing to its excellent stress resistance, low resource requirements, and high yield potential [1]. Its production scale is second only to that of wheat (*Triticum aestivum*) and rice (*Oryza sativa*), and it is the most productive non-cereal food crop globally [2]. Potato cultivation in China began in the early 19th century, initially introduced as a staple food and disaster relief crop in southwest China [3,4]. Since the 21st century, potato production has continued to grow. By 2024, China’s potato harvest area had reached over 4.5 million hectares, with production exceeding 94 million tons [5]. Special attention has been given to the colored potato germplasm resources formed by the natural mutation of common cultivars, whose periderm (tuber skin) and perimedullary tissue (tuber flesh) exhibit characteristic colors such as red, blue, purple, and black [6]. Colored potatoes not only exhibit phenotypic diversity but also contain anthocyanin levels ranging from 10 to 70 mg per 100 g FW—3 to 4 times higher than those of traditional varieties. While traditional varieties possess antioxidant capacities equivalent to 930 to 1380 mg Trolox equivalents per kg FW, colored varieties surpass these values by 2.5 to 3 times [7].

Anthocyanins are a class of water-soluble phenolic pigments belonging to the flavonoid group, widely distributed in the cell sap of various plant organs [8,9]. Six of the most common anthocyanins have been identified in potatoes. Pelargonidin-3-(p-coumaroyl-rutinoside)-5-glucoside is the primary anthocyanin in red-fleshed potatoes, while petunidin-3-(p-coumaroyl-rutinoside)-5-glucoside is the predominant anthocyanin in purple-fleshed potatoes [10]. Their biosynthetic pathway begins with phenylalanine, which is converted into 4-coumaroyl-CoA via the phenylpropanoid pathway [11], followed by the sequential generation of intermediate products through the flavonoid synthesis pathway, and finally the formation of anthocyanins via specific modifications [12]. In potatoes, this process is directly regulated by structural genes such as *PAL*, *CHS*, *CHI*, *F3H*, *DFR*, *F3′H*, *F3′5′H*, *ANS*, and *UFGT* while also being coregulated by modifying enzymes like acyltransferases [13]. Additionally, numerous transcription factors associated with potato anthocyanin biosynthesis have been identified, including MYB, bHLH, WKRY, WD40, and others. For example, *StMYBATV* competitively binds to *StbHLH1* to regulate potato anthocyanin biosynthesis, while StMYB3 negatively regulates tuber anthocyanin biosynthesis [14]. *StWRKY13* activates the activity of *StCHS*, *StF3H*, *StDFR*, and *StANS*, thereby promoting anthocyanin biosynthesis in potato tubers [15]. Although the biosynthetic pathway of anthocyanin glycosides has been studied in depth, its regulatory mechanisms in different plants still require further clarification. Especially in herbaceous crops like potatoes, genetic research is limited due to the lack of sufficient mutant resources.

As a dual-purpose crop serving as both a staple food and vegetable [16], potatoes not only provide traditional nutrients, such as starch (approximately 18–21%), protein (2–3%), and various minerals and vitamins, but are also rich in bioactive compounds including phenolic compounds [17], glycoside alkaloids [18], and dietary fiber [19]. These components confer multiple physiological benefits, including antioxidant and anti-inflammatory effects, the regulation of gut microbiota, and improvement in metabolic health [16,19,20]. Research confirmed that anthocyanins in colored potatoes hold significant potential for preventing alcoholic liver damage. One study established a chronic alcoholic liver injury model using 64 three-week-old Kunming mice (equal numbers of males and females). Mice were administered anthocyanins extracted from “Heijingang” potatoes (primarily containing petunidin-3-coumarosyl-5-glucoside) via oral gavage combined with three anthocyanin doses (2, 5, 10 mg/kg/day) over a 5-week period. The results demonstrated that this anthocyanin effectively alleviated alcohol-induced oxidative stress and liver damage [21]. Field observations revealed that colored potatoes were significantly more resistant to late blight than common varieties, which may be closely associated with the bacteriostatic activity of anthocyanin glycosides [22]. Phenylpropanoid compounds belong to the class of plant-induced antimicrobial substances. When plants encounter biotic stresses such as pathogen infection, the phenylpropanoid biosynthesis pathway within them is rapidly activated. PAL serves as the central key enzyme in this metabolic pathway. It catalyzes the deamination of phenylalanine to produce cinnamic acid, thereby mediating the biosynthesis of downstream phenolic compounds and ultimately enhancing the plant’s inherent antimicrobial defense capabilities [23]. Colored potatoes exhibit excellent light and heat stability, high yields, environmental adaptability, and low production costs. Their anthocyanins can be used as natural colorants in baked goods and beverages and also serve as natural preservatives in food applications [24]. Among these types of potatoes, Purple Majesty exhibited the highest anthocyanin content at 113.19 mg/100 g FW [25]. Given the coregulatory relationship between the anthocyanin biosynthesis pathway and defense/metabolic pathways, anthocyanin phenotypes can assist in the indirect screening of resistance-related traits (such as late blight resistance) [26].

Transcriptomes reflect the types and quantities of genes in cells, revealing the molecular mechanisms underlying physiological and biochemical processes [27]. RNA-seq, based on the Illumina platform, is widely used for transcript structure analysis, differential gene expression detection, alternative splicing (AS) identification, and transcript abundance quantification [28]. However, to overcome the limitations of short read lengths and improve sequencing accuracy, the third-generation sequencing technology single-molecule real-time (SMRT) sequencing has emerged [29,30]. SMRT sequencing achieves an average read length of over 10 kb (up to 60 kb) [31], enabling full-length transcriptome analysis that compensates for the shortcomings of second-generation sequencing [32]. Currently, SMRT sequencing is widely applied in the full-length transcriptome analysis of plants, particularly in AS event detection, significantly improving isoform identification sensitivity [33,34,35]. Thus, SMRT sequencing has distinct advantages over RNA-seq in AS analysis.

AS is the process by which a single precursor mRNA is spliced into multiple mature mRNAs so that the same gene produces multiple different mature mRNAs, which ultimately encode different proteins [36]. In organisms, five main types of AS exist, which are exon skipping (ES), alternative 5′ splice site (A5), alternative 3′ splice site (A3), intron retention (IR), and mutually exclusive exon (MEE) [37]. Studies have shown that alternative splicing plays a key role in many processes such as stress response [38,39,40], circadian regulation [41], and growth and development in plants. For example, the interaction between *CcMYB6-1* and *CcbHLH1* enhances the activity of the *CcF3H* and *CcDFR* promoters. The AS of *CcbHLH1* prevents it from co-activating the anthocyanin biosynthesis pathway with *CcMYB6-1*, leading to white cornflowers [42,43,44]. Different splicing variants of *CsbHLH133* respond to multiple stress conditions through specific regulatory patterns, thereby modulating the biosynthesis of geraniol in tea plants [45]. The AS of *CA10g11690* in chili peppers contributes to purple exocarp development [46], and the AS of *CsTT8* in the fourth exon in blood oranges forms Δ15-TT8, which may downregulate TT8 expression and affect fruit pigmentation [47]. In tea plants, the expression levels of genes including *C4H*, *CHS*, *FLS*, *F3′5′H*, *PAL*, *UFGT*, *ANR*, *ANS*, *F3H*, *4CL1*, *C3H*, *CHI*, and *DFR* and their AS transcripts were validated. The results indicate that the AS transcripts of *C4H1*, *FLS1*, and *PAL2* are key transcripts regulating anthocyanin biosynthesis in tea plants [48]. These studies strongly suggest that AS events are critical for the accumulation of anthocyanins in plants. Although some AS events have been identified during plant developmental stages, the regulatory mechanisms behind these events still need to be explored in depth.

Colored potatoes hold significant research value, yet the post-transcriptional regulatory mechanisms governing anthocyanin accumulation remain incompletely elucidated. This study’s significance resides in integrating SMRT sequencing and RNA-seq to analyze three different-colored potatoes across three developmental stages (tuber development stage, tuber enlargement stage, tuber harvest stage). Through SSR locus analysis, lncRNA characterization, and open reading frame (ORF) prediction, we identify differentially expressed genes and AS-related genes in anthocyanin biosynthesis—notably validating that StB3-like’s three AS-derived transcripts differentially regulate anthocyanin accumulation. This study fills a research gap in the role of alternative splicing within the potato anthocyanin biosynthesis regulatory pathway, clarifies a key post-transcriptional regulatory mechanism, and lays a solid foundation for further research on potato anthocyanin molecular mechanisms.

## 2. Results

### 2.1. Construction and Annotation of Full-Length Transcriptome of Potato

SMRT sequencing was performed separately on white (F01) and colored (F02) potato cultivars. After quality control, clean data volumes of 17.07 GB and 23.36 GB were obtained for F01 and F02 (Table S1). Based on cyclic common sequence (CCS) analysis, 365,197 and 566,802 CCSs were identified in the F01 and F02 samples, respectively (Table S2). The CCSs were categorized into full-length sequences and non-full-length sequences by detecting whether they contained 5′ primers, 3′ primers, and poly(A) tail structures. Among them, 309,043 and 481,798 full-length non-chimeric (FLNC) sequences were identified in F01 and F02, which accounted for 84.62% and 85.00% of the total number of corresponding CCSs, respectively (Table 1). After correcting the obtained FLNC sequences, 75,864 high-quality transcripts and 4330 low-quality transcripts were obtained in F01, and 102,209 high-quality transcripts and 3378 low-quality transcripts were obtained in F02 (Table 2). Subsequently, the corrected consensus sequences were aligned with the reference genome using GMAP (Genomic Mapping and Alignment Program), redundant transcripts were removed using cDNA_Cupcake (https://github.com/Magdoll/cDNA_Cupcake/wiki, accessed on 25 November 2025), and finally 38,663 and 46,249 non-redundant transcript sequences were obtained in the F01 and F02 samples, respectively. Alternative splicing analysis was performed on the de-redundant transcripts, and 15,270 and 16,852 loci were detected in the F01 and F02 samples, respectively.

### 2.2. Functional Annotations for New Transcripts

We utilized Clusters of Orthologous Groups (COG), Gene Ontology (GO), Kyoto Encyclopedia of Genes and Genomes (KEGG), the euKaryotic Ortholog Groups (KOG), Protein family (Pfam), Swiss-Prot Protein Sequence (Swiss-Prot), eggNOG, and NCBI Non-Redundant Protein Database (NR) for the functional annotation of proteins encoded by new transcripts. The annotation results showed that 17,840 (42.35%) new transcripts were annotated in the COG database, 33,582 (79.72%) in the GO database, 19,959 (47.38%) in the KEGG database, 27,266 (64.72%) in the KOG database, 30,703 (72.88%) in the Swiss-Prot database, 39,755 (94.37%) in the eggNOG database, and 42,065 (99.86%) in the NR database (Table S3). Combining the eight databases, a total of 8395 novel transcripts were co-annotated (Figure S1). KOG analysis showed that the 27,266 annotated novel transcripts were classified into 25 functional categories. Among them, the three categories with the highest percentage were General function prediction only; Signal transduction mechanisms; and Posttranslational modification, protein turnover, and chaperones (Figure S2). A total of 33,582 annotations were found in the GO enrichment analysis. The 33,582 annotated novel transcripts in the GO enrichment analysis were mainly enriched in different cellular components (CCs), molecular functions (MFs), and biological processes (BPs). Specifically, at the cellular component level, new transcripts were mainly related to cells and organelles; at the biological process level, they were mainly related to metabolic processes and cellular processes; at the molecular function level, the pathways were closely related to protein binding and catalytic activity (Figure S3). KEGG pathway enrichment analysis indicated that the most significantly enriched pathways for the new transcripts were Starch and sucrose metabolism, Protein processing in the endoplasmic reticulum, and the Spliceosome (Figure S4).

### 2.3. Prediction of CDSs, lncRNAs, and SSRs

TransDecoder software (v.3.0.0) was used to predict potential coding regions based on transcriptome sequences, and open reading frame (ORF) annotations were predicted for novel transcripts identified in the variable splicing analysis. This study predicted a total of 42,026 ORFs, including 36,002 complete ORFs. The protein sequences encoded by the predicted complete ORFs ranged in length from 100 to 2300 bp (Figure S5). Among them, proteins with lengths of 100–200 amino acids were the most abundant (8586, 23.85%), followed by proteins with lengths of 200–300 amino acids (7029, 19.52%) (Table S4).

**Figure 1:**
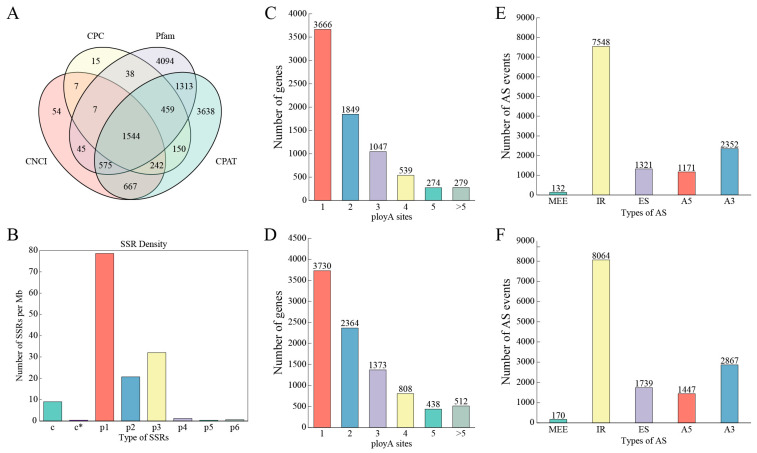
Predicted lncRNAs, SSRs, APA sites, and AS events based on PacBio sequencing data analysis. (**A**): Number of lncRNAs screened using CNCI, CPC, pfam, and CPAT. (**B**): Distribution of detected SSR types. c* represents the effective number of compound SSRs. (**C**): Distribution of number of polyadenylation sites in F01 sample. (**D**): Distribution of number of polyadenylation sites in F02 sample. (**E**): Statistics on number of alternative splicing events occurring in F01 sample. (**F**): Statistics on number of alternative splicing events occurring in F02 sample.

Long-stranded noncoding RNA (lncRNA) usually refers to RNA molecules that are greater than 200 nt in length and similar in structure to mRNA but do not have the ability to code for proteins [49]. Studies have shown that lncRNAs play important roles in various biological processes such as epigenetic regulation, transcriptional regulation, and post-transcriptional regulation. In this study, four methods, namely CPC, CNCI, Pfam, and CPAT, were combined for lncRNA prediction, and a total of 1544 lncRNA transcripts were identified (Figure 1A). Based on their genomic localization, these lncRNAs were classified into the following: intergenic lncRNAs (840), antisense lncRNAs (103), intronic lncRNAs (80), and sense lncRNAs (461) (Figure S6).

Simple sequence repeat (SSR) markers are widely used in the fields of plant genetic map construction, variety identification, genetic diversity analysis, and molecular marker-assisted selection because of their strong polymorphism, good repeatability, and high stability. In this study, 67,025 sequences (total length 148,382,563 bp) with a length greater than 500 bp were analyzed in terms of SSRs, and a total of 22,777 SSR sites were detected. Among them, 17,540 sequences contained at least one SSR site; among these 17,540 sequences, 3920 sequences contained more than one SSR site. The detected SSRs can be categorized into seven groups according to the type of repeat unit: Mono-nucleotide (13,270), Di-nucleotide (3804), Tri-nucleotide (5316), Tetra-nucleotide (201), Penta-nucleotide (52), Hexa-nucleotide (134), and compound SSRs (1560). The statistics showed that single-base repeat types were the most abundant, followed by three- and two-base repeat types, and five-base repeat types were the least abundant (Figure 1B, Table S5).

### 2.4. Identification of Alternative Polyadenylation (APA) and AS

The post-transcriptional processing of pre-mRNA into mature mRNA primarily includes 5′ end capping, 3′ end poly(A) tail addition, and intron splicing. Notably, the positional heterogeneity of the 3′ end poly(A) tail (variable poly(A) sites) can influence the binding of RNA-binding proteins to mRNA, thereby regulating RNA splicing and translation processes. This study analyzed the 3′ ends of transcripts based on PacBio sequencing data. The results showed that in the F01 sample, there were 3666 transcripts containing a single poly(A) site, while there were 279 transcripts containing five or more poly(A) sites (Figure 1C). In the F02 sample, there were 3730 transcripts with a single poly(A) site and 512 transcripts with five or more poly(A) sites (Figure 1D).

To investigate the effect of AS on anthocyanin accumulation in potatoes, this study performed alternative splicing analysis on 38,663 and 46,249 non-redundant transcripts. The results showed that the two groups of samples detected 15,270 and 16,852 gene loci, respectively, including 969 and 1270 new gene loci and 22,743 and 26,751 new transcripts, respectively. The alternative splicing types in the two samples were consistent, ranked in descending order by frequency as follows: intron retention (IR), alternative 3′ splice site (A3), exon skipping (ES), alternative 5′ splice site (A5), and mutually exclusive exon (MEE). Among these, the white variety detected a total of 12,524 AS events, including 132 MEE, 7548 IR, 1321 ES, 1711 A5, and 2353 A3 events (Figure 1E). The colored variety detected 14,287 AS events, including 170 MEE, 8064 IR, 1739 ES, 1447 A5, and 2867 A3 events (Figure 1F).

### 2.5. Transcription Factor Prediction

**Figure 2:**
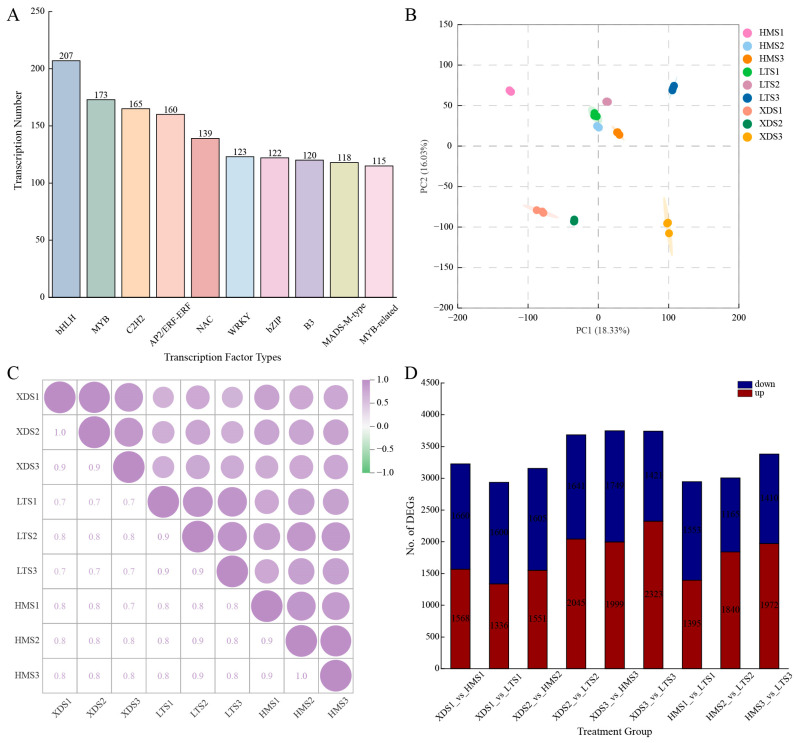
Characterization of short-read transcriptomic data. Values represent mean of three biological replicates. (**A**): Distribution of transcription factor types. (**B**): Principal component analysis of FPKM data collected from potato tubers at three developmental stages. (**C**): Pearson correlation analysis of potato tubers collected at three developmental stages. (**D**): Number of differentially expressed genes at three developmental stages.

Using iTAK software (v.1.5) for transcription factor prediction, a total of 2767 transcription factors (TFs) were identified, belonging to 68 different families. Among these, the bHLH family had the largest number of members (207), followed by the MYB family (173), C2H2 family (165), AP2/ERF-ERF family (160), NAC family (139), WRKY family (123), and bZIP family (122) (Figure 2A). Additionally, nine TF families had member counts between 50 and 120, specifically including the B3, MADS-M-type, MYB-related, C3H, GARP-G2-like, GRAS, HB-HD-ZIP, C2C2-GATA, and LOB families.

### 2.6. Short-Read Transcriptomic Correction for Full-Length Transcriptomics

RNA-seq was performed on 27 samples from three different-colored cultivars at three developmental stages using the Illumina platform. After quality control filtering to remove low-quality sequences, high-quality clean reads were obtained. The Q30 score was approximately 93%, and the GC content was approximately 44%, indicating good sequencing data quality (Table S6). Subsequently, the clean reads were aligned to the potato full-length transcriptome (FL transcriptome) reference sequence, and gene expression levels were estimated using the FPKM (Fragments Per Kilobase of transcript per Million mapped reads) method. To assess the relationships between samples, we performed principal component analysis (PCA) based on the FPKM expression data. The PCA results showed that all biological replicate samples clustered closely together, indicating high correlation among them and good experimental reproducibility. Samples from the three cultivars and three different developmental stages exhibited a clear separation trend (Figure 2B). These results suggest that developmental stage is the primary factor influencing transcriptomic changes associated with potato tuber color.

### 2.7. Differential Gene Analysis

**Figure 3:**
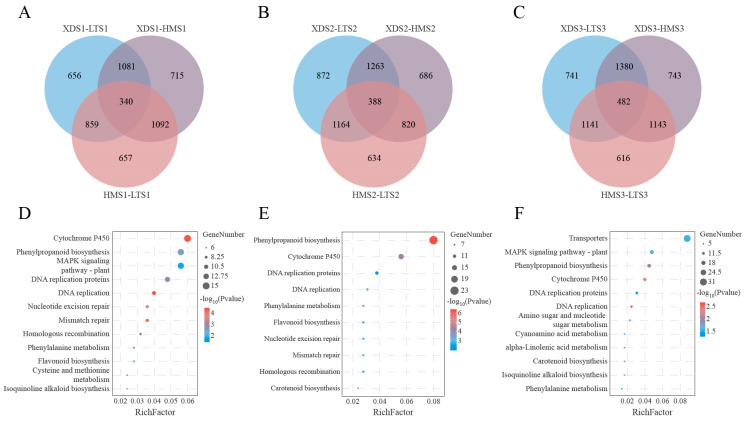
The analysis of differentially expressed genes (DEGs). (**A**–**C**): Venn diagrams showing the overlap of DEGs among the three potato varieties at the three tuber development stages. (**D**–**F**): The KEGG pathway analysis of DEGs among the three potato varieties at the three tuber development stages.

To validate the reliability of the differentially expressed gene (DEG) (log_2_FC > 1, FDR < 0.05) analysis results, Pearson correlation analysis was performed on each sample in this study. The results showed that the coefficient of determination (R^2^) between the three biological replicate samples under each experimental condition was greater than 0.7, indicating high consistency among biological replicates and providing a stable and reliable data foundation for subsequent DEG analysis (Figure 2C). Through systematic analysis, a total of 9350 DEGs were identified in the three potato clones. This study found that the number of DEGs showed a significant increasing trend as tuber development progressed. Specifically, the number of upregulated differentially expressed genes in the three clonal lines at the three developmental stages was 1336–1568, 1551–2045, and 1972–2323, while the corresponding numbers of downregulated differentially expressed genes were 1553–1660, 1165–1641, and 1410–1749, respectively (Figure 2D). Additionally, when comparing yellow and colored potato clones, a total of 4904 genes showed significant differential expression, with 1081, 1263, and 1380 differentially expressed genes in the S1, S2, and S3 developmental stages, respectively (Figure 3A–C). Intergroup comparisons showed that the differences between the XDS3 and HMS3 sample groups were the most significant, with a total of 3748 differentially expressed genes detected (including 1999 upregulated genes and 1749 downregulated genes), followed by the XDS3 and LTS3 sample groups, with 3744 differentially expressed genes identified (including 2323 upregulated genes and 1421 downregulated genes) (Figure 2D). The KEGG pathway enrichment analysis of differentially expressed genes showed that, in the S1 developmental stage, differentially expressed genes were significantly enriched in the cytochrome P450 metabolic pathway, plant MAPK signaling pathway, and phenylpropanoid biosynthesis pathway (Figure 3D); at the S2 stage, they were primarily enriched in the cytochrome P450 metabolic pathway and the phenylpropanoid biosynthesis pathway (Figure 3E); by the S3 stage, the plant MAPK signaling pathway and the phenylpropanoid biosynthesis pathway became the most significantly enriched pathways (Figure 3F).

### 2.8. Expression Patterns of Flavonoid Biosynthesis Genes in Different Cultivars

**Figure 4:**
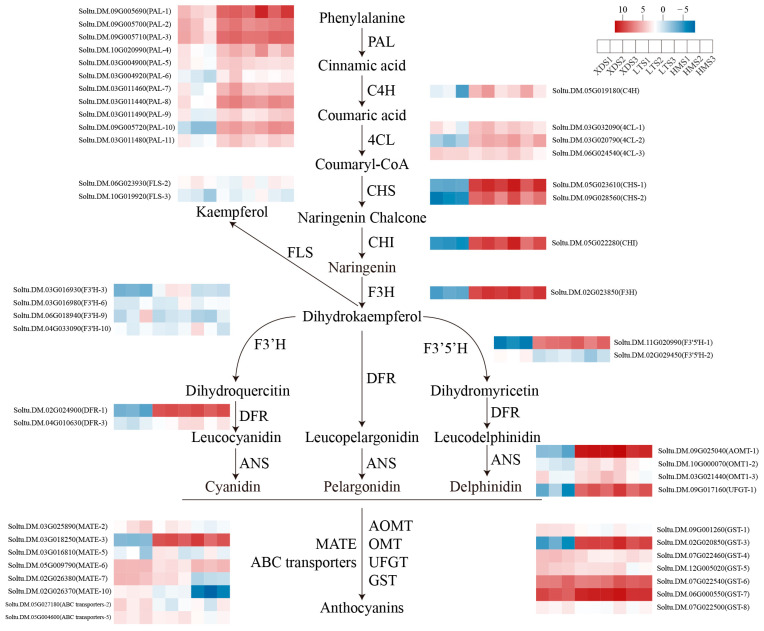
Gene expression of anthocyanin-related genes at different developmental stages across three varieties. Columns represent all samples; rows represent genes. Blue to red represents log_2_-transformed FPKM values. PAL, phenylalanine ammonia-lyase; C4H, trans-cinnamic acid 4-mono-hydroxylase; 4CL, 4-coumaroyl-CoA lyase; CHS, chalcone synthase; CHI, chalcone isomerase; FLS, flavonol synthase; F3H, flavone 3-hydroxylase; F3′H, flavonoid-3-hydroxylase; F3′5′H, flavonoid-3′,5′-hydroxylase; ABC transporter, ATP-binding cassette transporter; GST, glutathione S-transferase; UFGT, UDP-glucose flavone 3-O-glucosyltransferase; MATE, multidrug and toxin extruder; AOMT, flavone 3′,5′-methyltransferase.

To further elucidate the expression patterns of key genes in the anthocyanin biosynthesis pathway during different developmental stages of three potato cultivars, this study analyzed the expression profiles of genes associated with this pathway (Figure 4, Table S7). In the phenylpropanoid pathway upstream of anthocyanin synthesis, 11 differentially expressed phenylalanine ammonia-lyase (*PAL*) genes were detected, all of which exhibited high expression levels in colored potato cultivars. Cinnamic acid is converted into 4-coumaroyl-CoA via catalysis by cinnamic acid 4-hydroxylase (*C4H*) and 4-coumaroyl-CoA ligase (*4CL*). One *C4H* gene and three *4CL* genes showed significantly upregulated expression in colored potatoes. After entering the flavonoid synthesis pathway, two chalcone synthase genes (*CHS*) and one chalcone isomerase gene (*CHI*) associated with chalcone synthesis were all upregulated in colored potatoes. Concurrently, the high expression level of the flavanone 3-hydroxylase gene (*F3H*) in colored potatoes is presumed to promote the accumulation of dihydrokaempferol in their tubers. In the core pathway of anthocyanin biosynthesis, dihydroflavonol 4-reductase (*DFR*) reduces dihydrokaempferol, dihydroquercetin, and dihydromyricetin to colorless anthocyanins, which are subsequently catalyzed by anthocyanin synthase (*ANS*) to form colored anthocyanin aglycones. Flavonol synthase (*FLS*) converts dihydrokaempferol into kaempferol (a precursor diverging from anthocyanin biosynthesis). Analysis revealed that two *DFR* genes and the UDP-glucose flavonoid 3-O glucosyltransferase (*UFGT*) gene were highly expressed in colored potatoes. This upregulation is presumed to drive the increased accumulation of cyanidin, delphinidin, and pelargonidin in tubers. Additionally, the high expression level of the F3H, flavonoid 3′-hydroxylase (*F3′H*), and flavonoid 3′,5′-hydroxylase (*F3′5′H*) genes in colored potatoes likely contributes significantly to the red coloration of their tuber flesh. Among anthocyanin modification- and transport-related genes, six multidrug and toxin extrusion transporter (*MATE*) genes, seven glutathione S-transferase (*GST*) genes, one flavonoid 3′-O-methyltransferase (*AOMT*) gene, two O-O-methyltransferase (*OMT*) genes, and two ABC transporter genes were identified. Among these, the expression levels of the *MATE-3*, *GST-3*, and *AOMT-1* genes were significantly higher in LT and HM than in XD. Specifically, *MATE-3* (*Soltu.DM.03G018250*) exhibited expression ranges of 250–400 in LT and 170–580 in HM, and those of *GST-3* (*Soltu.DM.02G020850*) ranged from 440 to 510 (LT) and 320 to 860 (HM), while *AOMT-1* (*Soltu.DM.09G025040*) showed the most pronounced upregulation, with expression ranges reaching 1260–1441 in LT and 720–1750 in HM.

**Figure 5:**
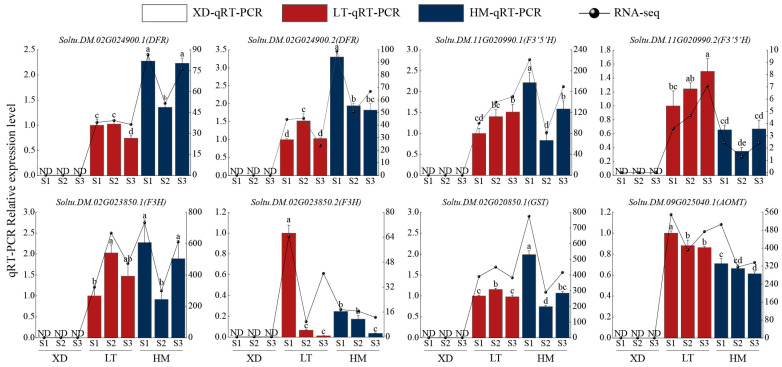
A quantitative expression analysis of five genes in Xindaping, Lingtianhongmei, and Heimeiren. XD represents Xindaping, LT represents Lingtianhongmei, and HM represents Heimeiren. Data represent the mean ± SE of three biological replicates. Different lowercase letters on the bar charts indicate significant differences among combinations (*p* < 0.05). ND, not detected.

To validate the reliability of RNA-seq data, five genes (*DFR*, *F3H*, *GST*, *F3*′*5*′*H*, *AOMT*) associated with anthocyanin biosynthesis were selected for qRT-PCR analysis. The results showed that although the expression trends in the qRT-PCR results differed from those in RNA-seq data, the overall expression trends were consistent. This indicates that the qRT-PCR results are significantly correlated with RNA-seq data, demonstrating the reliability of RNA-seq data (Figure 5).

### 2.9. Analysis of Alternative Splicing in Potato Anthocyanin Biosynthesis-Related Genes and Transcription Factors

#### 2.9.1. AS in Structural Genes

To clarify the role of AS in anthocyanin accumulation during potato growth and development, we first analyzed AS events in structural genes involved in anthocyanin biosynthesis (Table S8). The results revealed that 17 out of 88 structural genes exhibited alternative splicing, accounting for 19% of the total genes. Among these, *F3H (Soltu.DM.02G023850)*, *DFR-1 (Soltu.DM.02G024900)*, *MATE-3 (Soltu.DM.03G018250)*, *CHI (Soltu.DM.05G022280)*, *CHS-2 (Soltu.DM.09G028560)*, and *F3′5′H-1* (*Soltu.DM.11G020990*) each produced two transcripts. Expression trends showed lower levels in XD cultivars and increased expression in HM and LT cultivars. *OMT1-2* (*Soltu.DM.10G000070*) also produces two transcripts with distinct expression patterns: *Soltu.DM.10G000070.1* shows low expression in the XD cultivar but high expression in HM and LT, while *Soltu.DM.10G000070.2* exhibits no significant differences across the three cultivars. For *4CL-3* (*Soltu.DM.06G024540*), *Soltu.DM.06G024540.1* exhibited low expression in the LT cultivar and high expression in the XD and HM cultivars during the S1 stage. During the S3 stage, it showed low expression in the HM cultivar and high expression in the XD and LT cultivars. while no significant differences were observed among the three cultivars during the S2 stage. However, *Soltu.DM.06G024540.2* exhibited the same expression trend across all cultivars. No obvious trends were observed for the remaining structural genes.

#### 2.9.2. AS in Transcription Factors

**Figure 6:**
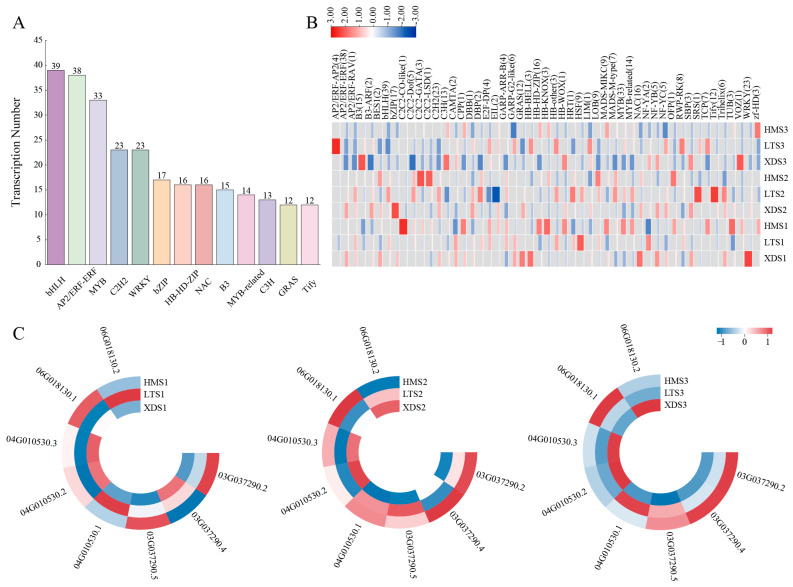
Expression patterns of transcription factor families in transcripts. (**A**): Distribution of transcription factors in transcripts. (**B**): Heatmap of transcription factor families across three potato varieties at different developmental stages. Values underwent log2 transformation and row normalization. (**C**): Heatmaps of three groups of transcription factors with different transcripts and highest differential expression in XD, LT, and HM.

Transcription factors (TFs) play a key regulatory role in gene expression regulatory networks. In this study, we identified a total of 403 differentially expressed TFs (log_2_FC > 2, FDR < 0.01), belonging to 51 different families. Among these, the most abundant family was the bHLH family (39 members), followed by the AP2/ERF-ERF family (38 members), the MYB family (33 members), and the C2H2 and WRKY families, which were tied for fourth place (23 members each) (Figure 6A). Further analysis revealed that the FPKM values of most transcription factors were higher in LT and HM tuber flesh. Notably, the NF-YB and SBP families exhibited high expression levels in XL at all three developmental stages while showing low expression levels in the tuber flesh of both LT and HM; HB-other TFs were specifically highly expressed in LT tuber flesh across all three stages while showing low expression in both the XD and HM cultivars; C2C2-GATA, C2C2-LSD, HB-HD-ZIP, HB-KNOX, and MADS-MIKC TFs maintained high expression levels in the tuber flesh of HM. Additionally, SRS and Tify TFs were highly expressed only in tuber flesh tissue at the LTS2 stage, while GRAS and WRKY TFs peaked at the S1 stage in XD tuber flesh. Notably, MYB TFs maintained stable high expression levels in the tuber flesh of the LT and HM cultivars across all three stages (Figure 6B).

To further explore the contribution of AS to anthocyanin regulation, we analyzed AS patterns among TFs. The results revealed that 33 out of 360 transcription factors belonging to 51 families exhibited alternative splicing, with 29 of these being differentially expressed (FPKM > 1, log_2_FC > 2). Several TF families showed significant expression divergence among alternative transcripts (Table S9). The AP2/ERF-ERF family member *Soltu.DM.03G037290* produced three transcripts with distinct expression patterns: *Soltu.DM.03G037290.2* showed higher expression in the HM cultivar, and *Soltu.DM.03G037290.4* showed progressively increasing expression in the S3 stage across cultivars XD, LT, and HM, while *Soltu.DM.03G037290.5* exhibited rising expression levels in the order XD, LT, HM across the three stages. The B3 family *Soltu.DM.04G010530* (*StB3-like*) produced three transcripts: *Soltu.DM.04G010530.1* exhibited low expression in the XD cultivar but significantly increased in the LT and HM cultivars, and *Soltu.DM.04G010530.2* and *Soltu.DM.04G010530.3* exhibited the highest expression in the XD cultivar, followed by the HM cultivar, and the lowest in the LT cultivar. Within the HB-HD-ZIP family, *Soltu.DM.06G018130* comprises two transcripts. *Soltu.DM.06G018130.1* exhibited divergent expression trends across developmental stages in the three cultivars: expression increased in the order LT, XD, HM during the S1 and S2 stages but reversed to XD, LT, HM during the S3 stage. Conversely, *Soltu.DM.06G018130.2* exhibited higher expression levels in the LT cultivar during the S1 stage, with lower expression in the XD and HM cultivars. During the S2 and S3 stages, its expression increased in the XD cultivar while decreasing in the other two cultivars (Figure 6C). These results indicate that alternative splicing-generated transcripts regulate anthocyanin accumulation across different cultivars and developmental stages.

### 2.10. Different Transcripts Result in Varying Anthocyanin Content in Tobacco Leaves

**Figure 7:**
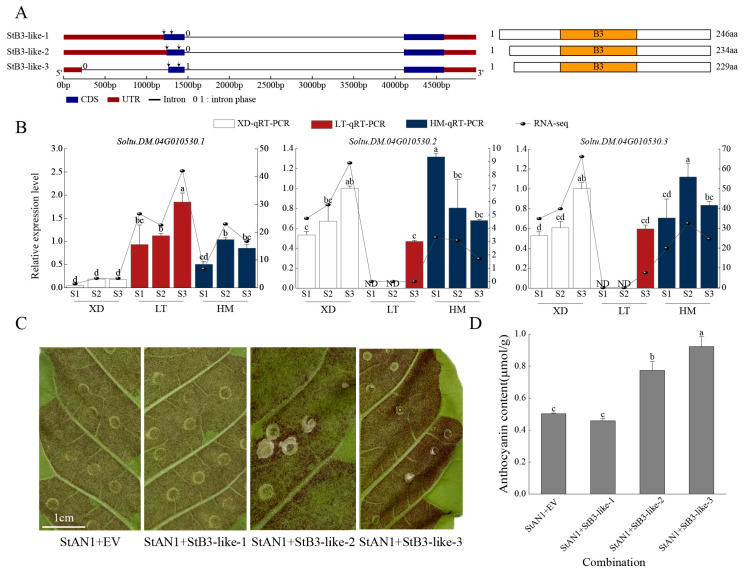
The preliminary functional characterization of three StB3-like transcripts in anthocyanin synthesis via tobacco transient coloration experiments. (**A**): The left panel shows the exons and introns of three transcripts, with arrows indicating the positions of qRT-PCR primers. The right panel displays the domains and amino acid deletion sites within the transcripts. (**B**): The quantitative expression analysis of the three transcripts in tobacco cultivars Xindaiping, Lingtianhongmei, and Heimeiren. (**C**): The phenotypes of tobacco leaves injected with four combinations: *StAN1 + EV*, *StAN1 + StB3-like-1*, *StAN1 + StB3-like-2*, and *StAN1 + StB3-like-3*. (**D**): Anthocyanin content in tobacco leaves was measured after the co-injection of *StB3-like-1*, *StB3-like-2*, and *StB3-like-3* with AN1. Data represent the mean ± standard error of three biological replicates and were analyzed by a one-way ANOVA. Different lowercase letters on the bar charts indicate significant differences among combinations (*p* < 0.05).

The *StB3-like* gene was selected for further functional validation, and *StB3-like-1* represents the full-length transcript of *StB3-like*, which is predicted to encode 246 amino acids. The first exon of *StB3-like-1* undergoes alternative splicing to form *StB3-like-2*, encoding 234 amino acids. *StB3-like-2* then undergoes exon-specific splicing to generate *StB3-like-3*, which encodes 229 amino acids (Figure 7A). Previous research has confirmed that *StAN1 (Soltu.DM.10G020850)*, as a positive regulator of anthocyanin biosynthesis, significantly promoted anthocyanin accumulation when overexpressed in tobacco [50]. To validate the expression levels of the selected transcripts, qRT-PCR analysis was performed to determine the expression levels of the three *StB3-like* transcripts in the cultivars XD, LT, and HM. The results confirmed that expression trends aligned closely with FPKM values, supporting the reliability of the identified transcripts (Figure 7B). To investigate the functional specificity of each *StB3-like* transcript, transient overexpression assays were conducted in tobacco (variety Nc89). First, we individually injected *StB3-like-1*, *StB3-like-2*, and *StB3-like-3* into tobacco plants and found that they did not affect anthocyanin accumulation (Figure S7). Four combinations were tested, including *StAN1 + EV*, *StAN1 + StB3-like-1*, *StAN1 + StB3-like-2*, and *StAN1 + StB3-like-3* coexpression combinations. *StAN1 + EV* was injected into one side of the tobacco leaf as a control, while StAN1 and each coexpression combination were injected into the other side (Figure 7C). After culturing the injected bacterial solution for 3~5 days, it was observed that compared to the *StAN1 + EV* treatment group, the leaf color of the *StAN1 + StB3-like-1* combination showed visibly lighter pigmentation compared with the control, while the leaf color of the *StAN1 + StB3-like-2* and *StAN1 + StB3-like-3* combinations significantly darkened. A quantitative analysis of anthocyanins confirmed that leaves injected with the *StAN1 + StB3-like-2* and *StAN1 + StB3-like-3* combinations exhibited significantly higher anthocyanin glycoside content compared to leaves injected with the *StAN1 + StB3-like-1* combination (Figure 7D). These results indicate that *StB3-like* alternative transcripts individually have no effect on anthocyanin biosynthesis. However, when co-injected with *AN1*, *StB3-like-2* and *StB3-like-3* function as positive regulators, while *StB3-like-1* appears to exert only a weak or neutral influence.

**Figure 8:**
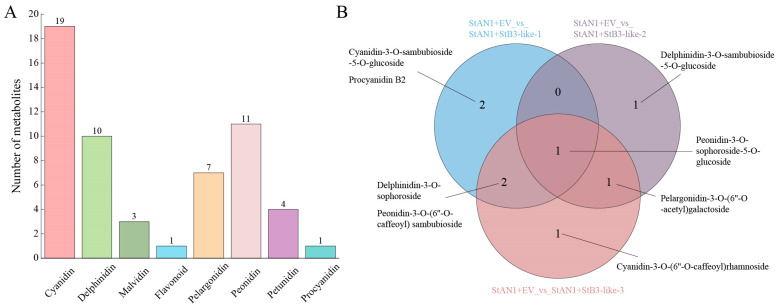
Anthocyanin metabolomic analysis of leaves injected with different transcripts. (**A**): Classification of all anthocyanins detected in EV, StB3-like-1, StB3-like-2, and StB3-like-3. (**B**): Venn diagram comparing three combinations.

To further characterize the anthocyanin profiles associated with each transcript, we performed a targeted quantification of anthocyanin components in the infiltrated tobacco leaves using UPLC-MS/MS. Orthogonal partial least squares discriminant analysis (OPLS-DA) confirmed high data quality and reproducibility across biological replicates (Figure S8). A total of eight anthocyanin classes were detected, with cyanidin and peonidin derivatives being the most abundant, while delphinidin, pelargonidin, and other classes were present at relatively lower levels (Figure 8A). Following co-injection with StAN1, comparative analysis revealed distinct differential metabolite profiles for each StB3-like transcript compared to the EV control (Figure 8B). Specifically, five differential metabolites were identified for both StB3-like-1 and StB3-like-3, whereas only three were altered in StB3-like-2. Notably, Peonidin-3-O-sophoroside-5-O-glucoside was a common differential metabolite across all three transcripts. In summary, the three StB3-like transcripts differentially regulate anthocyanin metabolism in tobacco by distinctly influencing the biosynthesis and accumulation of specific anthocyanin compounds.

**Figure 9:**
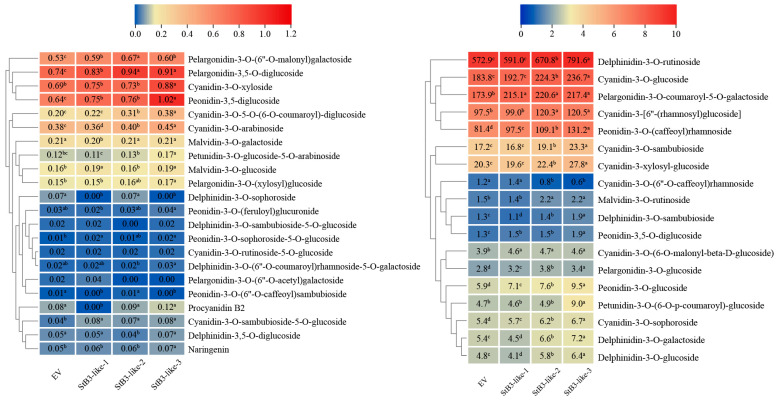
Heatmap of differential metabolites (log_2_FC > 2) in leaves injected with different transcripts. Data represent absolute quantification of anthocyanin metabolites, with values averaged from three biological replicates. Different lowercase letters above numbers indicate significant differences between combinations (*p* < 0.05).

To investigate the effects of different transcripts on anthocyanin synthesis, we analyzed changes in anthocyanin metabolite levels (Figure 9). Among the detected metabolites, Delphinidin-3-O-rutinoside, Cyanidin-3-O-glucoside, Pelargonidin-3-O-coumaroyl-5-O-galactoside, Cyanidin-3-[6″-(rhamnosyl) glucoside, and Peonidin-3-O-(caffeoyl) rhamnoside exceeded 100 μg/g. Among these, Delphinidin-3-O-rutinoside, Cyanidin-3-O-glucoside, and Peonidin-3-O-(caffeoyl) rhamnoside exhibited the highest concentrations in *StB3-like-3*, followed by *StB3-like-2*, while *StB3-like-1* showed the lowest levels. The Cyanidin-3-[6″-(rhamnosyl) glucoside content in *StB3-like-2* and *StB3-like-3* reached 1.2-fold and 1.3-fold that of StB3-like-1, respectively. Two metabolites (Cyanidin-3-O-sambubioside and Cyanidin-3-xylosyl-glucoside) showed high expression levels in *StB3-like-3*, with concentrations ranging from 16 to 30 μg/g. Eleven metabolites ranged in concentration from 1 to 10 μg/g: eight of these (including Delphinidin-3-O-glucoside, Delphinidin-3-O-galactoside, Cyanidin-3-O-sophoroside, Petunidin-3-O-(6-O-p-coumaroyl)-glucoside, Peonidin-3-O-glucoside, Pelargonidin-3-O-glucoside, Peonidin-3,5-O-diglucoside, Delphinidin-3-O-sambubioside) exhibited the highest content in *StB3-like-3*; Malvidin-3-O-rutinoside was highly expressed in both *StB3-like-2* and *StB3-like-3*, whereas Cyanidin-3-O-(6″-O-caffeoyl) rhamnoside showed the highest expression level in *StB3-like-1*. Twenty-two metabolites had concentrations below 1 μg/g, with one-third exhibiting relatively high levels in *StB3-like-3*.

## 3. Discussion

Colorful potatoes are widely cultivated due to their exceptionally high nutritional value. Investigating the accumulation mechanism of anthocyanins in potatoes is of significant importance for the practical application of colorful potatoes in production and daily life. Currently, existing studies utilize SMRT sequencing combined with RNA-Seq data analysis to investigate the regulatory gene network governing the biosynthesis of anthocyanin compounds in potatoes. Potato tubers serve as crucial organs for nutrient absorption, storage, and transport in plants, playing an indispensable role in plant growth, development, and stress responses. Under potato heat stress conditions, SMRT sequencing yielded 245,298 FLNC reads and 58,747 high-quality full-length transcripts [51]. Through PacBio sequencing, a total of 11,665 complete open reading frames were predicted in potato seedlings [52]. Based on SMRT sequencing, 5955 long noncoding RNAs and 700 AS events were identified in Populus plants [53]. In this study, the PacBio sequencing of white and colored potato varieties yielded over 40 GB of clean data and 178,073 FLNC reads, enabling the comprehensive identification of 42,065 transcripts, 36,002 complete CDSs, 2061 TFs, 1544 lncRNAs, and extensive structural features including SSRs and poly-A sites. Among them, lncRNAs may regulate anthocyanin biosynthesis through cis/trans-acting mechanisms, while SSRs can serve as molecular markers linked to anthocyanin-related genes, providing valuable genomic resources for further exploring their functional implications in potato anthocyanin metabolism.

This study integrated RNA-seq data from three developmental stages of different potato cultivars with the FL transcriptome of this study to investigate the DEGs and their enriched pathways associated with anthocyanin accumulation in potato tubers. The results showed that the number of DEGs was higher in the XDS3 vs. HMS3 and XDS3 vs. LTS3 comparison groups compared to the first and second stages. During potato development, biological pathways are primarily enriched in the cytochrome P450 pathway, MAPK signaling pathway—plant, phenylpropanoid biosynthesis pathway, DNA replication protein pathway, DNA replication pathway, and transporter pathway. Relevant studies have confirmed that the phenylpropanoid biosynthesis pathway participates in the accumulation process of anthocyanins [54,55]. Furthermore, the enrichment in the transporter pathway aligns with the conclusion that “ATP-binding cassette transporters are key regulators of anthocyanin accumulation in potato tubers” [56]. The cytochrome P450 pathway, meanwhile, is closely associated with the modification of secondary metabolites during anthocyanin synthesis [57]. Pathway enrichment patterns varied across developmental stages. Stage S1 showed enrichment in the cytochrome P450 pathway, MAPK signaling pathway—plant pathway, phenylpropanoid biosynthesis pathway, DNA replication protein pathway, and DNA replication pathway. Stage S2 showed enrichment only in the cytochrome P450 pathway, phenylpropanoid biosynthesis pathway, DNA replication protein pathway, and DNA replication pathway. Stage S3 demonstrated enrichment in all six pathways. This likely reflects the coordinated regulatory mechanism governing anthocyanin synthesis and transport during tuber maturation [58].

AS enhances the coding capacity of the genome by generating multiple transcripts through the utilization of multiple splicing sites within genes. Currently, research on AS in plants primarily focuses on areas such as salt stress [59], light signal transduction [60], fruit development [61], high-temperature stress, and drought stress [62]. Studies have shown that mutations in the 5′ splicing site of the second intron of the *DFR* (dihydroflavonol-4-reductase) gene in Solanaceae plants affect anthocyanin accumulation [63]. In the Sichuan pepper varieties “Rongchang Wucai” (WC) and “Zhuye” (ZY), there are 143,122 unique coding sequences, 105,465 SSRs, 20,145 TFs, and 4719 lncRNAs, with 142,829 transcripts successfully annotated [64]. In grape berries, the AS variant *VvMYBA1-L* of the *VvMYBA1* gene causes the premature termination of the encoded protein; the overexpression of *VvMYBA1-L* delays the accumulation of flesh color, while the overexpression of *VvMYBA1* promotes the accumulation of flesh color [65]. In this study, 12,524 AS events were identified in F01, and 14,287 AS events were identified in F02. After screening for differentially expressed transcripts from the transcripts obtained from the alternative splicing analysis, three groups of genes were found to have corresponding transcripts. These differentially expressed genes associated with anthocyanin synthesis via AS events warrant further investigation in future studies.

Transcription factors, as key components of gene regulatory networks, play a central role in plants’ responses to both biotic and abiotic stresses. During their functional processes, transcription factors undergo post-transcriptional modifications, with alternative splicing mechanisms being one of the most extensively studied. In soybean, the overexpression of *GmNTL9.m1* resulted in wilting and chlorotic symptoms under sodium bicarbonate stress, whereas the overexpression of *GmNTL9.m2* conferred significant tolerance [66]. In tea plants, the full-length *CsJAZ1-1* and variant *CsJAZ1-2* interact with *CsMYC2* to inhibit anthocyanin biosynthesis, while *CsJAZ1-3* relieves this inhibition through a competitive mechanism [67]. In rapeseed, *BnaPAP2.A7-744* positively regulates purple leaf pigmentation, whereas *BnaPAP2.A7-910* and *BnaPAP2.A7-395* have no effect on it [68]. The AS of CiFD in citrus forms two splicing variants (CiFDα and CiFDβ); CiFDβ directly binds to the promoter of CiAP1 to promote flowering, while CiFDα exerts its function by forming an FAC complex that binds to the CiAP1 promoter. The B3 transcription factor family comprises four subfamilies: LAV, RAV, ARE, and REM [69]. Strawberry *FaRAV1* promotes anthocyanin accumulation by activating the *FaMYB10* promoter and structural genes involved in anthocyanin biosynthesis [70]. Apple *MdARF13* inhibits anthocyanin accumulation by directly binding to the *MdDFR* promoter [71]. The Arabidopsis REM subfamily gene *VRN1* (vernalization 1) participates in regulating plant flowering [72,73]. In this study, we focused on investigating the roles of different transcripts of the B3 family transcription factor *StB3-like* in anthocyanin biosynthesis. The results showed that the co-injection of *StB3-like-2* and *StB3-like-3* with *StAN1* markedly promoted anthocyanin accumulation compared with the control, whereas *StB3-like-1* exerted no apparent effect. Metabolomic analysis further revealed that different transcripts specifically regulate the accumulation of multiple anthocyanin-related metabolites. For instance, *StB3-like-3* specifically accumulates compounds such as Petunidin-3-O-(6-O-p-coumaroyl)-glucoside. Although some transcripts exhibited primer cross-reactivity in qRT-PCR detection, the distinct metabolite profiles clearly support *StB3-like-2* and *StB3-like-3* as independent transcripts. These findings suggest that different *StB3-like* transcripts may participate in regulating the anthocyanin biosynthesis pathway by specifically controlling metabolite accumulation.

Beyond the B3 family, we also identified AS events in transcription factors such as bHLH, MYB, and AP2/ERF-ERF. For example, the AS of *CmbHLH2* in chrysanthemum generates two variants: only the full-length *CmbHLH2^Full^* interacts with *CmMYB6* to promote anthocyanin biosynthesis, while the *CmbHLH2^Short^* variant is non-functional [74]. The AS of *VaMYB108* in grape produces *VaMYB108^normal^* and *VaMYB108^short^*, both induced by low temperature and capable of mutually upregulating each other’s expression [28]. However, whether the AS of these transcription factors affects anthocyanin accumulation in potato requires further verification.

### Scope and Limitations

Functional validation was performed in tobacco, a heterologous system. Although the results provide strong evidence for the regulatory role of StB3-like transcripts, further potato-specific validation (e.g., stable transformation, gene silencing) is required to confirm their causal roles in tuber anthocyanin accumulation. Additionally, the mechanisms underlying AS regulation remain to be explored.

## 4. Materials and Methods

### 4.1. Preparation of Plant Materials

**Figure 10:**
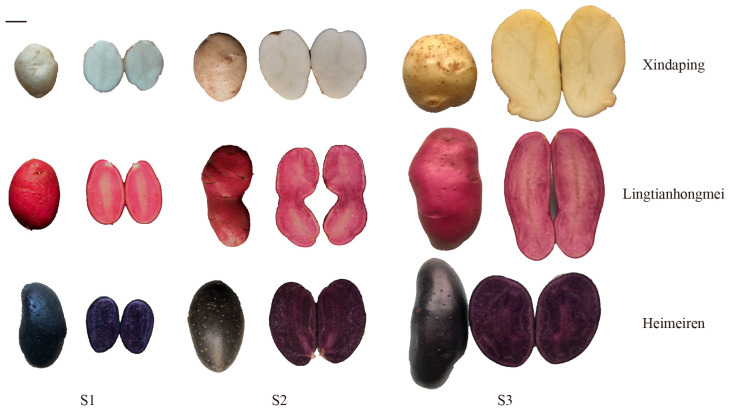
Tuber phenotypes of Xindaping (XD), Lingtianhongmei (LT), and Heimeiren (HM) across three growth stages (S1: tuber development stage; S2: tuber enlargement stage; S3: tuber harvest stage). Scale bar = 2 cm.

The potato clonal varieties used in this study included the yellow-skinned Xindaping, the red-skinned Lingtianhongmei, and the purple-skinned Heimeiren, all cultivated at the Dingxi Academy of Agricultural Sciences in Gansu Province, where the annual average temperature is 6.3 °C, the annual average sunshine duration is 2500.1 h, the soil type is yellow loam, and irrigation was maintained at 75–80% of field capacity (Figure 10). Potato tubers were harvested at 100 days after planting (S1, tuber development stage), 130 days (S2, tuber enlargement stage), and 150 days (S3, tuber harvest stage). An average of five tubers per plant were collected for each cultivar at each stage, with three biological replicates for all samples. The collected samples were immediately frozen in liquid nitrogen and then stored at −80 °C for subsequent use.

### 4.2. RNA Isolation, Library Preparation, and SMRT Sequencing

RNA was extracted from potato tubers using the TaKaRa Polysaccharide-Polyphenol RNA Extraction Kit (TaRaKa, Beijing, China). Its integrity and concentration were assessed via 1% agarose gel electrophoresis and Nanodrop ND-2000 (Thermo Fisher Scientific Inc., Shanghai, China), respectively. Subsequently, the SMARTer™ PCR cDNA Synthesis Kit (TaRaKa, Beijing, China) was used to synthesize full-length cDNA from mRNA. The full-length cDNA was then amplified by PCR (20 cycles). The full-length cDNA underwent end repair, followed by ligation with SMRT dumbbell-shaped adapters. Nucleic acid exoenzymes were used to digest the cDNA, resulting in a sequencing library. The obtained clean reads (Q20 > 90%) were mapped to the S.tuberosum reference genome (PGSC_DM_v6.1, http://solanaceae.plantbiology.msu.edu/pgsc_download.shtml, accessed on 16 November 2025) using HISAT2 (v2.0.1, https://daehwankimlab.github.io/hisat2/, accessed on 18 November 2025). DESeq2 was used for differential expression analysis between samples. Genes with log_2_FC > 1 and FDR < 0.05 were considered significantly differentially expressed genes (DEGs). DEGs involved in anthocyanin accumulation were screened based on KEGG functional annotation [75].

### 4.3. Identification of Novel Transcripts (SSR), Prediction of Long Noncoding RNAs (lncRNAs), and Sequence Annotation

New transcripts longer than 500 bp were screened and analyzed using MISA (http://pgrc.ipk-gatersleben.de/misa/, accessed on 20 November 2025) for SSRs [76]. MISA identifies seven types of SSRs by analyzing transcript sequences: single-base repeats, double-base repeats, triple-base repeats, quadruple-base repeats, quintuple-base repeats, sextuple-base repeats, and mixed SSRs.

TransDecoder (v.3.0.0) identifies reliable potential coding regions from transcript sequences based on open reading frame (ORF) length, log-likelihood score, and the alignment of amino acid sequences with protein domains in the Pfam database [77]. Four encoding potential analysis methods—CPC analysis, CNCI analysis, Pfam protein domain analysis, and CPAT analysis—were used to predict lncRNAs in newly discovered transcripts [78]. Astalavista (v.3.2) was used to identify five types of alternative splicing in the samples: ES, A3, A5, IR, and MEE.

The sequences of the newly identified transcripts were aligned with the NR, Swiss-Prot, GO, COG, KOG, Pfam, and KEGG databases using BLAST (v.2.2.26), resulting in the annotation of transcripts. Transcription factor prediction was performed using iTAK (v.1.5).

### 4.4. Quantitative Real-Time PCR (qRT-PCR) Analysis

RNA was extracted from the samples using an RNA extraction kit (Cat. No. 9769, TaKaRa, Beijing, China). The RNA samples were reverse-transcribed into cDNA using the FastKing gDNA Dispelling RT SuperMix kit (TianGen, Beijing, China). The SuperReal PreMix Plus kit (TianGen, Beijing, China) was used according to the manufacturer’s instructions, followed by qRT-PCR on the Bio-Rad CFX 96 system (BioRad Laboratories, Hercules, CA, USA). The *StEF-1α* (AB061263) gene was used as an internal control. Each assay was performed in triplicate, and gene expression levels were analyzed using the 2^−ΔΔCt^ method [79]. The specific primers used in this experiment are detailed in Table S10.

### 4.5. Functional Validation of Candidate Genes via Transient Expression in Tobacco

In this study, the three transcripts of *Soltu.DM.04G010530* were then cloned into the pNC-Green-SK vector and transformed into the Agrobacterium strain GV3101. For the transient coloration experiment, Nc89 was used as the experimental material. After 3–5 days of injection, the coloration of the tobacco leaves was observed, and samples were collected for subsequent experiments.

### 4.6. Anthocyanin Content Determination

The anthocyanin content in tobacco was determined based on the structural properties of anthocyanins under different pH conditions. Anthocyanins exhibit a maximum absorption peak at 530 nm when pH = 1, while at pH = 4.5, anthocyanins convert to colorless chalcones, resulting in no absorption peak at 530 nm. The determination was conducted as follows: Tobacco leaves were ground into powder. A total of 0.1 g of the sample was weighed and mixed with 1 mL of extraction solution. After thorough homogenization, it was transferred to an EP tube. The extraction solution was diluted to 1 mL. The solution was extracted at 60 °C for 30 min, then centrifuged at 12,000 rpm for 10 min at room temperature. The supernatant was aspirated for analysis [80]. The anthocyanin content in the sample was determined by measuring the absorbance values at 530 nm and 700 nm under different pH conditions (BC1385, Solarbio, Beijing, China).

### 4.7. Metabolite Analysis

Metware Biotechnology (Wuhan, China) performed the metabolomic analysis for this study. Tobacco leaves were freeze-dried using a vacuum freeze dryer (Scientz-100F, Ningbo, China) and ground into powder using a ball mill (MM 400, Retsch, Haan, North Rhine-Westphalia, Germany). A total of 50 mg of each sample was weighed and dissolved in 500 μL of 50% methanol aqueous solution (containing 0.1% hydrochloric acid). Each sample was weighed three times as biological replicates. The mixture was vortexed for 5 min, sonicated for 5 min, and centrifuged at 4 °C and 12,000 rpm for 3 min. The supernatant was aspirated, and the procedure was repeated once. The two supernatants were combined and filtered using a 0.22 μm microporous membrane (Millipore, Billerica, MA, USA) for filtration. Metabolites were identified using ultra-performance liquid chromatography (UPLC, ExionLC™ AD, SCIEX, Framingham, MA, USA) coupled with tandem mass spectrometry (MS/MS, QTRAP^®^ 6500+, SCIEX, Framingham, MA, USA) (UPLC-MS/MS).

Orthogonal partial least squares discriminant analysis (OPLS-DA) was performed on identified metabolites using R (v4.2.2). Significant differences were determined in metabolites between groups based on an absolute log_2_FC (fold change) > 2 and variable importance in projection (VIP) ≥ 1 [81].

### 4.8. Statistical Analysis

All experiments were performed in triplicate, with the results presented as the mean ± standard error (SE). Data analysis was conducted using SPSS (v.26.0) for a one-way analysis of variance (ANOVA) and Duncan’s multiple range test with *p* < 0.05.

## 5. Conclusions

This study aimed to elucidate the molecular mechanisms underlying pigment biosynthesis in potato tubers by integrating second- and third-generation transcriptomic sequencing technologies across different developmental stages. Transcriptomic analysis revealed abundant AS events and numerous novel transcripts, highlighting the complexity of gene regulation in potatoes. Notably, the *StB3-like* gene family exhibits distinct functional differentiation. Transient expression experiments confirmed that only *StB3-like-2* and *StB3-like-3* (but not *StB3-like-1*) significantly enhance anthocyanin accumulation when coexpressed with *StAN1*, validating their specific regulatory roles in pigment deposition. Collectively, this study deepens our understanding of transcriptional mechanisms linking disease resistance and pigmentation, providing valuable molecular markers and functional candidate genes for breeding nutritionally rich, visually appealing, and disease-resistant potato.

## Figures and Tables

**Table 1:** Table of full-length sequence statistics.

Samples	Number of CCS	Number of Undesired Primer Reads	Number of Filtered Short Reads	Number of Full-Length Non-Chimeric Reads	Full-Length Non-Chimeric Percentage (FLNC%)
F01	365,197.00	30,108.00	0.00	309,043.00	84.62%
F02	566,802.00	46,413.00	0.00	481,798.00	85.00%

**Table 2:** Table of clustering statistics results.

Samples	Number of Consensus Isoforms	Average Read Length of Consensus Isoforms	Number of Polished High-Quality Isoforms	Number of Polished Low-Quality Isoforms
F01	80,535.00	2189.00	75,864.00	4330.00
F02	105,748.00	1904.00	102,209.00	3378.00

## Data Availability

Data are contained within the article and Supplementary Materials.

## Supplementary Materials

The following supporting information can be downloaded at https://www.mdpi.com/article/10.3390/plants15030514/s1.

## References

[1] Stokstad E. (2019). The New Potato. Science.

[2] Camire M.E., Kubow S., Donnelly D.J. (2009). Potatoes and Human Health. Crit. Rev. Food Sci. Nutr..

[3] Haverkort A.J., Struik P.C. (2015). Yield Levels of Potato Crops: Recent Achievements and Future Prospects. Field Crops Res..

[4] Tang J.Z., Wang J., Fang Q.X., Wang E.L., Yin H., Pan X.B. (2018). Optimizing Planting Date and Supplemental Irrigation for Potato across the Agro-Pastoral Ecotone in North China. Eur. J. Agron..

[5] FAO (Food and, Agrciulture Organization of the United Nations) Statistics of Production: Crops. https://www.fao.org/faostat/en/.

[6] Samiha T.K., Rahman M.A., Islam S., Jalal N., Islam A., Nahiyan A.S.M. (2024). Exploring Potato Diversity: A Comprehensive Genetic and Phenotypic Analysis of Quantitative and Qualitative Traits. Czech J. Genet. Plant Breed..

[7] Zhou F., Wang T., Zhang B.L., Zhao H.F. (2018). Addition of Sucrose during the Blueberry Heating Process Is Good or Bad? Evaluating the Changes of Anthocyanins/Anthocyanidins and the Anticancer Ability in HepG-2 Cells. Food Res. Int..

[8] Xu Z.S., Feng K., Que F., Wang F., Xiong A.S. (2017). A MYB Transcription Factor, DcMYB6, Is Involved in Regulating Anthocyanin Biosynthesis in Purple Carrot Taproots. Sci. Rep..

[9] Zhou H., Lin-Wang K., Wang F.R., Espley R.V., Ren F., Zhao J.B., Ogutu C., He H.P., Jiang Q., Allan A.C. (2019). Activator-Type R2R3-MYB Genes Induce a Repressor-Type R2R3-MYB Gene to Balance Anthocyanin and Proanthocyanidin Accumulation. New Phytol..

[10] Liu Y., Tikunov Y., Schouten R.E., Marcelis L.F.M., Visser R.G.F., Bovy A. (2018). Anthocyanin Biosynthesis and Degradation Mechanisms in Solanaceous Vegetables: A Review. Front. Chem..

[11] Albert N.W., Lewis D.H., Zhang H., Irving L.J., Jameson P.E., Davies K.M. (2009). Light-Induced Vegetative Anthocyanin Pigmentation in *Petunia*. J. Exp. Bot..

[12] Wade H.K., Bibikova T.N., Valentine W.J., Jenkins G.I. (2001). Interactions within a Network of Phytochrome, Cryptochrome and UV-B Phototransduction Pathways Regulate Chalcone Synthase Gene Expression in *Arabidopsis* Leaf Tissue. Plant J..

[13] Jaakola L. (2013). New Insights into the Regulation of Anthocyanin Biosynthesis in Fruits. Trends Plant Sci..

[14] Liu Y.H., Li Y.M., Liu Z., Wang L., Lin-Wang K., Zhu J.Y., Bi Z.Z., Sun C., Zhang J.L., Bai J.P. (2023). Integrative Analysis of Metabolome and Transcriptome Reveals a Dynamic Regulatory Network of Potato Tuber Pigmentation. iScience.

[15] Zhang H.L., Zhang Z.H., Zhao Y.N., Guo D.L., Zhao X.J., Gao W., Zhang J.P., Song B.T. (2021). *StWRKY13* Promotes Anthocyanin Biosynthesis in Potato (*Solanum tuberosum*) Tubers. Funct. Plant Biol..

[16] Zaheer K., Akhtar M.H. (2016). Potato Production, Usage, and Nutrition—A Review. Crit. Rev. Food Sci. Nutr..

[17] Murniece I., Karklina D., Galoburda R., Santare D., Skrabule I., Costa H.S. (2011). Nutritional Composition of Freshly Harvested and Stored Latvian Potato (*Solanum tuberosum* L.) Varieties Depending on Traditional Cooking Methods. J. Food Compos. Anal..

[18] Milner S.E., Brunton N.P., Jones P.W., O’Brien N.M., Collins S.G., Maguire A.R. (2011). Bioactivities of Glycoalkaloids and Their Aglycones from Solanum Species. J. Agric. Food Chem..

[19] Raigond P., Ezekiel R., Raigond B. (2015). Resistant Starch in Food: A Review. J. Sci. Food Agric..

[20] McGill C.R., Kurilich A.C., Davignon J. (2013). The Role of Potatoes and Potato Components in Cardiometabolic Health: A Review. Ann. Med..

[21] Jiang Z.H., Chen C., Wang J., Xie W.Y., Wang M., Li X.S., Zhang X.Y. (2016). Purple Potato (*Solanum tuberosum* L.) Anthocyanins Attenuate Alcohol-Induced Hepatic Injury by Enhancing Antioxidant Defense. J. Nat. Med..

[22] Lachman J., Hamouz K. (2005). Red and Purple Coloured Potatoes as a Significant Antioxidant Source in Human Nutrition—A Review. Plant Soil Environ..

[23] Zhang W., Ma Y.F., Kang Y.C., Zhang R.Y., Wang Y., Chen Z.J., Yang X.Y., Jiao S.J., Wang X.X., Qin S.H. (2024). Transcriptome Analysis Reveals Various Genes Involved in the Regulation of Potato to Late Blight. Chem. Biol. Technol. Agric..

[24] Sampaio S.L., Lonchamp J., Dias M.I., Liddle C., Petropoulos S.A., Glamočlija J., Alexopoulos A., Santos-Buelga C., Ferreira I.C.F.R., Barros L. (2021). Anthocyanin-Rich Extracts from Purple and Red Potatoes as Natural Colourants: Bioactive Properties, Application in a Soft Drink Formulation and Sensory Analysis. Food Chem..

[25] Soare R., Dinu M., Babeanu C., Soare M. (2020). Evaluation and Comparison of Antioxidant Activity and Biochemical Compounds in Some Coloured Potato Cultivars. Plant Soil Environ..

[26] Beketova M.P., Chalaya N.A., Zoteyeva N.M., Gurina A.A., Kuznetsova M.A., Armstrong M., Hein I., Drobyazina P.E., Khavkin E.E., Rogozina E.V. (2021). Combination Breeding and Marker-Assisted Selection to Develop Late Blight Resistant Potato Cultivars. Agronomy.

[27] Han Y., Han Z., Lu Y., Han Z., Zhang J., Zhang J., Qiao H., He H. (2025). Expression and Analysis of *StNR* and *StNiRs*, Key Enzyme Genes of Nitrogen Assimilation in Potato (*Solanum tuberosum* L.) with Different Nitrogen Efficiencies. Czech J. Genet. Plant Breed..

[28] Hou Y.J., Li Q.Y., Zhou H.M., Kafle S., Li W.J., Tan L.S., Liang J., Meng L., Xin H.P. (2024). SMRT Sequencing of a Full-Length Transcriptome Reveals Cold Induced Alternative Splicing in *Vitis amurensis* Root. Plant Physiol. Biochem..

[29] Jia D., Wang Y.X., Liu Y.H., Hu J., Guo Y.Q., Gao L.L., Ma R.Y. (2018). SMRT Sequencing of Full-Length Transcriptome of Flea Beetle *Agasicles hygrophila* (Selman and Vogt). Sci. Rep..

[30] Sharon D., Tilgner H., Grubert F., Snyder M. (2013). A Single-Molecule Long-Read Survey of the Human Transcriptome. Nat. Biotechnol..

[31] Li W., Lee J., Yu S., Wang F.D., Lv W.Q., Zhang X., Li C.H., Yang J.L. (2021). Characterization and Analysis of the Transcriptome Response to Drought in *Larix kaempferi* Using PacBio Full-Length cDNA Sequencing Integrated with de Novo RNA-Seq Reads. Planta.

[32] Eid J., Fehr A., Gray J., Luong K., Lyle J., Otto G., Peluso P., Rank D., Baybayan P., Bettman B. (2009). Real-Time DNA Sequencing from Single Polymerase Molecules. Science.

[33] Abdel-Ghany S.E., Hamilton M., Jacobi J.L., Ngam P., Devitt N., Schilkey F., Ben-Hur A., Reddy A.S.N. (2016). A Survey of the Sorghum Transcriptome Using Single-Molecule Long Reads. Nat. Commun..

[34] Wang B., Tseng E., Regulski M., Clark T.A., Hon T., Jiao Y.P., Lu Z.Y., Olson A., Stein J.C., Ware D. (2016). Unveiling the Complexity of the Maize Transcriptome by Single-Molecule Long-Read Sequencing. Nat. Commun..

[35] Xu Z.C., Peters R.J., Weirather J., Luo H.M., Liao B.S., Zhang X., Zhu Y.J., Ji A.J., Zhang B., Hu S.N. (2015). Full-Length Transcriptome Sequences and Splice Variants Obtained by a Combination of Sequencing Platforms Applied to Different Root Tissues of *Salvia miltiorrhiza* and Tanshinone Biosynthesis. Plant J..

[36] Nilsen T.W., Graveley B.R. (2010). Expansion of the Eukaryotic Proteome by Alternative Splicing. Nature.

[37] Reddy A.S.N., Marquez Y., Kalyna M., Barta A. (2013). Complexity of the Alternative Splicing Landscape in Plants. Plant Cell.

[38] Wu Z., Liang J.H., Wang C.P., Ding L.P., Zhao X., Cao X., Xu S.J., Teng N.J., Yi M.F. (2019). Alternative Splicing Provides a Mechanism to Regulate LlHSFA3 Function in Response to Heat Stress in Lily. Plant Physiol..

[39] Seo P.J., Park M.-J., Park C.-M. (2013). Alternative Splicing of Transcription Factors in Plant Responses to Low Temperature Stress: Mechanisms and Functions. Planta.

[40] Zhu G.Z., Li W.X., Zhang F., Guo W.Z. (2018). RNA-Seq Analysis Reveals Alternative Splicing under Salt Stress in Cotton, *Gossypium davidsonii*. BMC Genom..

[41] Filichkin S.A., Mockler T.C. (2012). Unproductive Alternative Splicing and Nonsense mRNAs: A Widespread Phenomenon among Plant Circadian Clock Genes. Biol. Direct.

[42] Deng C.Y., Zheng X.F., Wang J.Y., Li Y.F., Li J.J., Lu M., Gao R.N., Ji C.Y., Hao Q.H., Dai S.L. (2024). Alternative Splicing of Activator *CcbHLH1* Gene Accounts for Anthocyanin Absence in White Cornflower. Ornam. Plant Res..

[43] Deng C., Wang J., Lu C., Li Y., Kong D., Hong Y., Huang H., Dai S. (2020). *CcMYB6-1* and *CcbHLH1*, Two Novel Transcription Factors Synergistically Involved in Regulating Anthocyanin Biosynthesis in Cornflower. Plant Physiol. Biochem..

[44] Zhang S., Ren Y., Zhao Q., Wu Y., Zhuo Y., Li H. (2023). Drought-Induced CsMYB6 Interacts with CsbHLH111 to Regulate Anthocyanin Biosynthesis in *Chaenomeles speciosa*. Physiol. Plant.

[45] Cheng L., Tu G.F., Ma H.C., Zhang K.Y., Wang X.Y., Zhou H.Z., Gao J.W., Zhou J., Yu Y.B., Xu Q.S. (2024). Alternative Splicing of *CsbHLH133* Regulates Geraniol Biosynthesis in Tea Plants. Plant J..

[46] Li N., Liu Y.B., Yin Y.X., Gao S.H., Wu F.Y., Yu C.Y., Wang F., Kang B.C., Xu K., Jiao C.H. (2023). Identification of *CaPs* Locus Involving in Purple Stripe Formation on Unripe Fruit, Reveals Allelic Variation and Alternative Splicing of R2R3-MYB Transcription Factor in Pepper (*Capsicum annuum* L.). Front. Plant Sci..

[47] Wang J.H., Xu R., Qiu S.P., Wang W.C., Zheng F. (2023). *CsTT8* Regulates Anthocyanin Accumulation in Blood Orange through Alternative Splicing Transcription. Hortic. Res..

[48] Chen L., Shi X., Nian B., Duan S., Jiang B., Wang X., Lv C., Zhang G., Ma Y., Zhao M. (2020). Alternative Splicing Regulation of Anthocyanin Biosynthesis in *Camellia sinensis* Var. Assamica Unveiled by PacBio Iso-Seq. G3 Genes Genomes Genet..

[49] Iyer M.K., Niknafs Y.S., Malik R., Singhal U., Sahu A., Hosono Y., Barrette T.R., Prensner J.R., Evans J.R., Zhao S. (2015). The Landscape of Long Noncoding RNAs in the Human Transcriptome. Nat. Genet..

[50] Liu Y.H., Lin-Wang K., Espley R.V., Wang L., Yang H.Y., Yu B., Dare A., Varkonyi-Gasic E., Wang J., Zhang J.L. (2016). Functional Diversification of the Potato R2R3 MYB Anthocyanin Activators AN1, MYBA1, and MYB113 and Their Interaction with Basic Helix-Loop-Helix Cofactors. J. Exp. Bot..

[51] Shang L.N., Zhou Y.H., Wen S.Q., Wang K., Li Y., Zhang M.H., Jian H.J., Lyu D.Q. (2023). Construction of Heat Stress Regulation Networks Based on Illumina and SMRT Sequencing Data in Potato. Front. Plant Sci..

[52] Yan C.C., Zhang N., Wang Q.Q., Fu Y.Y., Zhao H.Y., Wang J.J., Wu G., Wang F., Li X.Y., Liao H.J. (2022). Full-Length Transcriptome Sequencing Reveals the Molecular Mechanism of Potato Seedlings Responding to Low-Temperature. BMC Plant Biol..

[53] Li W.L., Liu Z.W., Feng H., Yang J.L., Li C.H. (2022). Characterization of the Gene Expression Profile Response to Drought Stress in *Populus ussuriensis* Using PacBio SMRT and Illumina Sequencing. Int. J. Mol. Sci..

[54] Wu X.J., Ma Y.H., Wang P.J., Wu J., Li N., Zhang Z.C., Xie R., Wang D., Nie H.S. (2024). Transcriptome Sequencing and Screening of Anthocyanin Related Genes in Purple Potato Tubers (*Solanum tuberosum* L.). BMC Genom..

[55] He M., Ma X., Zhou Y., Wang F., Fang G., Wang J. (2024). Combined Metabolome and Transcriptome Analyses Reveals Anthocyanin Biosynthesis Profiles Between Purple and White Potatoes. Int. J. Mol. Sci..

[56] Yang R., Huang S., Li D., Sun Y., Zhou G., Zhou D., Huang B. (2025). Transcriptomic and Targeted Metabolomic Analysis Identifies Genes Involved in Differential Anthocyanin Accumulation in Potato Tubers. Front. Plant Sci..

[57] Riveros-Loaiza L.M., Benhur-Cardona N., Lopez-Kleine L., Soto-Sedano J.C., Pinzón A.M., Mosquera-Vásquez T., Roda F. (2022). Uncovering Anthocyanin Diversity in Potato Landraces (*Solanum tuberosum* L. Phureja) Using RNA-Seq. PLoS ONE.

[58] Li M., Xiong Y.T., Yang X.Y., Gao Y.L., Li K.H. (2024). Transcriptomic and Metabolic Analysis Reveals Genes and Pathways Associated with Flesh Pigmentation in Potato (*Solanum tuberosum*) Tubers. Curr. Issues Mol. Biol..

[59] Li Y., Guo Q.H., Liu P., Huang J.G., Zhang S.Z., Yang G.D., Wu C.G., Zheng C.C., Yan K. (2021). Dual Roles of the Serine/Arginine-Rich Splicing Factor SR45a in Promoting and Interacting with Nuclear Cap-Binding Complex to Modulate the Salt-Stress Response in *Arabidopsis*. New Phytol..

[60] Li Y., Du Y.X., Huai J.L., Jing Y.J., Lin R.C. (2022). The RNA Helicase UAP56 and the E3 Ubiquitin Ligase COP1 Coordinately Regulate Alternative Splicing to Repress Photomorphogenesis in *Arabidopsis*. Plant Cell.

[61] Li Y.P., Dai C., Hu C.G., Liu Z.C., Kang C.Y. (2017). Global Identification of Alternative Splicing via Comparative Analysis of SMRT- and Illumina-Based RNA-Seq in Strawberry. Plant J..

[62] Sun M., Huang D.J., Zhang A.L., Khan I., Yan H.D., Wang X.S., Zhang X.Q., Zhang J., Huang L.K. (2020). Transcriptome Analysis of Heat Stress and Drought Stress in Pearl Millet Based on Pacbio Full-Length Transcriptome Sequencing. BMC Plant Biol..

[63] Wang X., Chen X.P., Luo S.X., Ma W., Li N., Zhang W.W., Tikunov Y., Xuan S.X., Zhao J.J., Wang Y.H. (2022). Discovery of a *DFR* Gene That Controls Anthocyanin Accumulation in the Spiny *Solanum* Group: Roles of a Natural Promoter Variant and Alternative Splicing. Plant J..

[64] Liu X.M., Tang N., Xu F., Chen Z.X., Zhang X., Ye J.B., Liao Y.L., Zhang W.W., Kim S.-U., Wu P.Y. (2022). SMRT and Illumina RNA Sequencing Reveal the Complexity of Terpenoid Biosynthesis in *Zanthoxylum armatum*. Tree Physiol..

[65] Gao L., Wang W., Li H.R., Li H., Yang Y.X., Zheng H., Tao J.M. (2023). Anthocyanin Accumulation in Grape Berry Flesh Is Associated with an Alternative Splicing Variant of VvMYBA1. Plant Physiol. Biochem..

[66] Liu X., Li M.L., Chen T., Zhang R., Wang Y.Y., Xiao J.L., Ding X.D., Zhang S.Z., Li Q. (2024). A Global Survey of Bicarbonate Stress-Induced Pre-mRNA Alternative Splicing in Soybean via Integrative Analysis of Iso-Seq and RNA-Seq. Int. J. Biol. Macromol..

[67] Zhu J.Y., Yan X.M., Liu S.R., Xia X.B., An Y.L., Xu Q.S., Zhao S., Liu L., Guo R., Zhang Z.L. (2022). Alternative Splicing of *CsJAZ1* Negatively Regulates Flavan-3-Ol Biosynthesis in Tea Plants. Plant J..

[68] Chen D.Z., Liu Y., Yin S., Qiu J., Jin Q.D., King G.J., Wang J., Ge X.H., Li Z.Y. (2020). Alternatively Spliced *BnaPAP2.A7* Isoforms Play Opposing Roles in Anthocyanin Biosynthesis of *Brassica napus* L.. Front. Plant Sci..

[69] Wei M.K., Li H., Wang Q., Liu R., Yang L.X., Li Q.Z. (2023). Genome-Wide Identification and Expression Profiling of B3 Transcription Factor Genes in *Populus alba × Populus glandulosa*. Front. Plant Sci..

[70] Zhang Z.Y., Shi Y.N., Ma Y.C., Yang X.F., Yin X.R., Zhang Y.Y., Xiao Y.W., Liu W.L., Li Y.D., Li S.J. (2020). The Strawberry Transcription Factor FaRAV1 Positively Regulates Anthocyanin Accumulation by Activation of *FaMYB10* and Anthocyanin Pathway Genes. Plant Biotechnol. J..

[71] Wang Y.C., Wang N., Xu H.F., Jiang S.H., Fang H.C., Su M.Y., Zhang Z.Y., Zhang T.L., Chen X.S. (2018). Auxin Regulates Anthocyanin Biosynthesis through the Aux/IAA–ARF Signaling Pathway in Apple. Hortic. Res..

[72] Levy Y.Y., Mesnage S., Mylne J.S., Gendall A.R., Dean C. (2002). Multiple Roles of *Arabidopsis* VRN1 in Vernalization and Flowering Time Control. Science.

[73] Sung S., Amasino R.M. (2004). Vernalization in *Arabidopsis thaliana* Is Mediated by the PHD Finger Protein VIN3. Nature.

[74] Lim S.-H., Kim D.-H., Jung J.-A., Lee J.-Y. (2021). Alternative Splicing of the Basic Helix–Loop–Helix Transcription Factor Gene *CmbHLH2* Affects Anthocyanin Biosynthesis in Ray Florets of Chrysanthemum (*Chrysanthemum morifolium*). Front. Plant Sci..

[75] Wang W.L., Liu Z., Qi Z.Y., Li Z.T., Zhu J.Y., Chen L.M., Li Y.M., Bi Z.Z., Yao P.F., Sun C. (2024). Integrated Transcriptomic and Metabolomic Analyses Reveal Transcriptional Regulatory Network for Phenolic Acid Biosynthesis in Potato Tubers. Food Biosci..

[76] Thiel T., Michalek W., Varshney R.K., Graner A. (2003). Exploiting EST Databases for the Development and Characterization of Gene-Derived SSR-Markers in Barley (*Hordeum vulgare* L.). Theor. Appl. Genet..

[77] Haas B.J., Papanicolaou A., Yassour M., Grabherr M., Blood P.D., Bowden J., Couger M.B., Eccles D., Li B., Lieber M. (2013). *De Novo* Transcript Sequence Reconstruction from RNA-Seq Using the Trinity Platform for Reference Generation and Analysis. Nat. Protoc..

[78] Ye J.B., Cheng S.Y., Zhou X., Chen Z.X., Kim S.U., Tan J.P., Zheng J.R., Xu F., Zhang W.W., Liao Y.L. (2019). A Global Survey of Full-Length Transcriptome of *Ginkgo biloba* Reveals Transcript Variants Involved in Flavonoid Biosynthesis. Ind. Crops Prod..

[79] Tang X., Zhang N., Si H., Calderón-Urrea A. (2017). Selection and Validation of Reference Genes for RT-qPCR Analysis in Potato under Abiotic Stress. Plant Methods.

[80] Hu W., Chen Y.L., Xu Z.Z., Liu L.Q., Yan D., Liu M.Y., Yan Q.D., Zhang Y.H., Yang L., Gao C.X. (2024). Natural Variations in the *Cis*-Elements of *GhRPRS1* Contributing to Petal Colour Diversity in Cotton. Plant Biotechnol. J..

[81] Liu Y.H., Li Y.M., Liu Z., Wang L., Bi Z.Z., Sun C., Yao P.F., Zhang J.L., Bai J.P., Zeng Y.T. (2023). Integrated Transcriptomic and Metabolomic Analysis Revealed Altitude-Related Regulatory Mechanisms on Flavonoid Accumulation in Potato Tubers. Food Res. Int..

