# Supplementary material for: Combined SMRT and Illumina RNA Sequencing Reveals the Alternative Splicing-Mediated Regulation of Anthocyanin Accumulation in Potato (Solanum tuberosum L.)
Source: Plants (Basel). 2026 Feb 6;15(3):514. doi: 10.3390/plants15030514 (PMC12899072; doi:10.3390/plants15030514)
Supplement: Supplementary file 1 [file plants-15-00514-s001.zip › Fig.S1-S8.pdf]

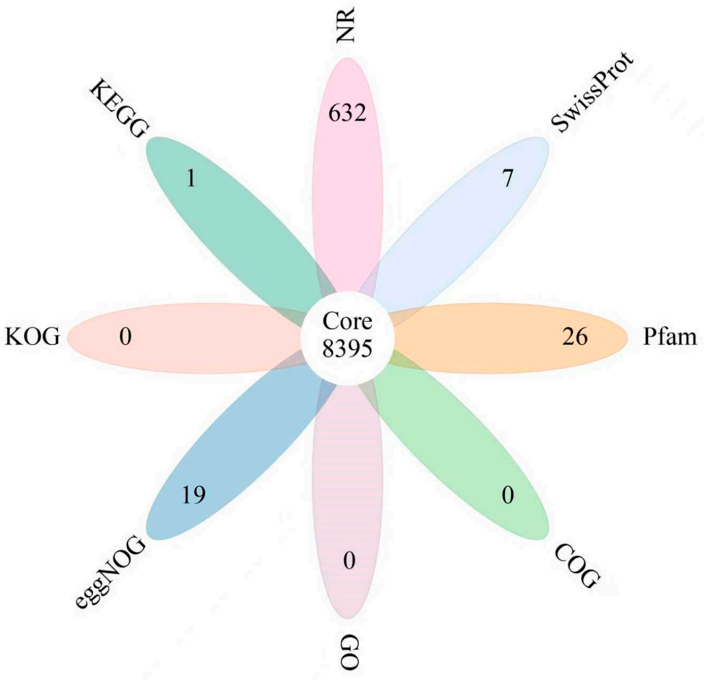

Figure S1. Transcripts annotated in the Nr, GO, KO, KEGG, KOG, Swissprot, eggNOG, and Pfam databases.

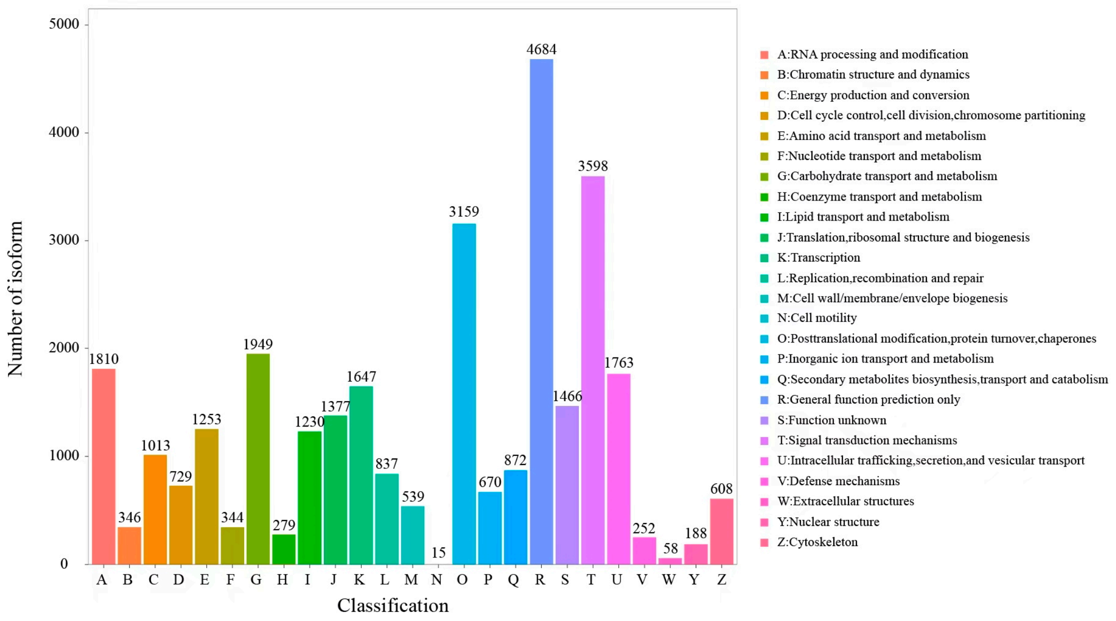

Figure S2. Functional annotation of new transcripts in the KOG database.

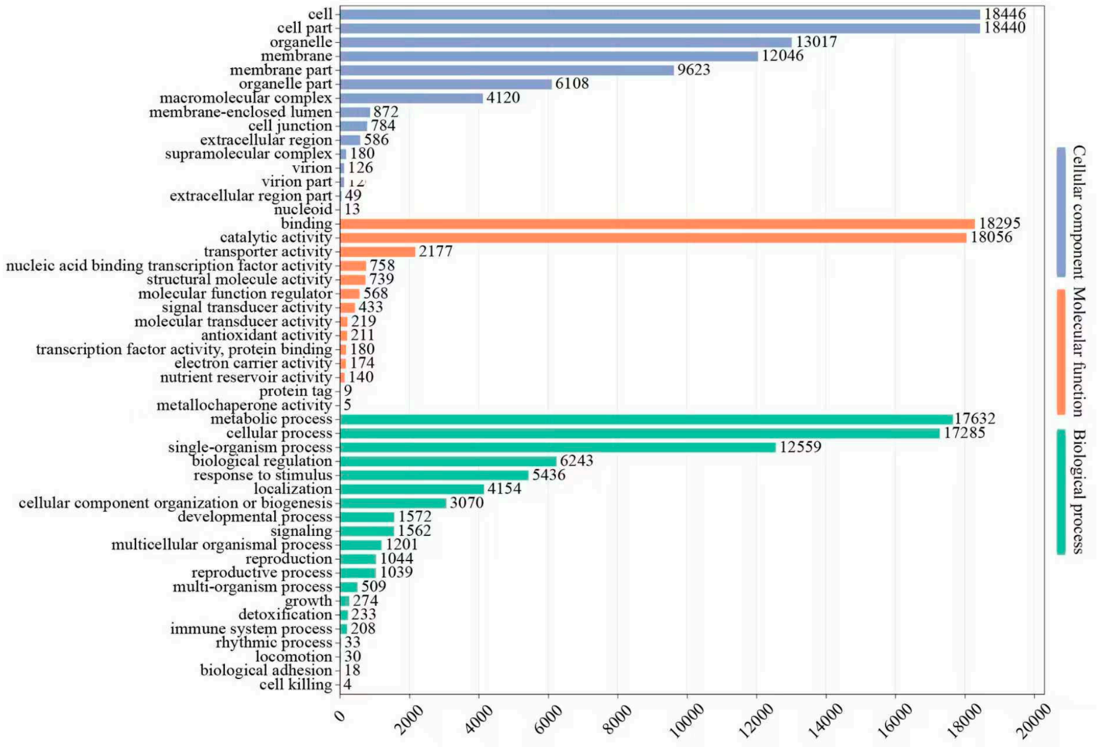

Figure S3. Functional annotation of new transcripts in the GO database.

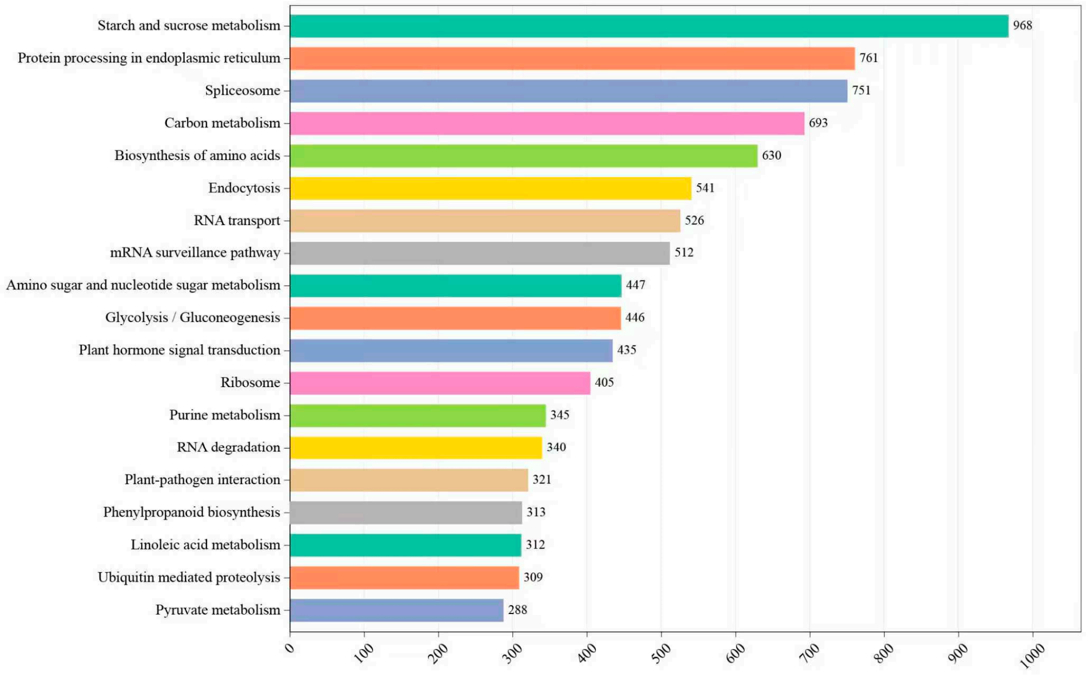

Figure S4. Functional annotation of new transcripts in the KEGG database.

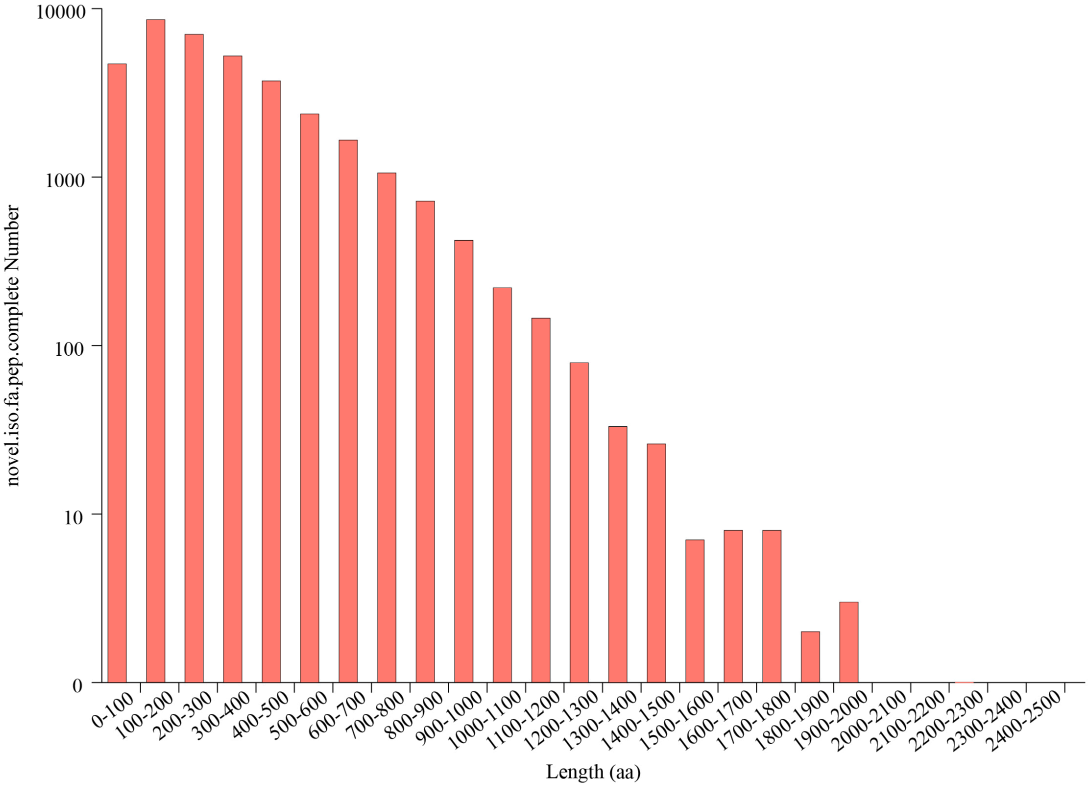

Figure S5. Predicted CDS-encoded protein length distribution diagram.

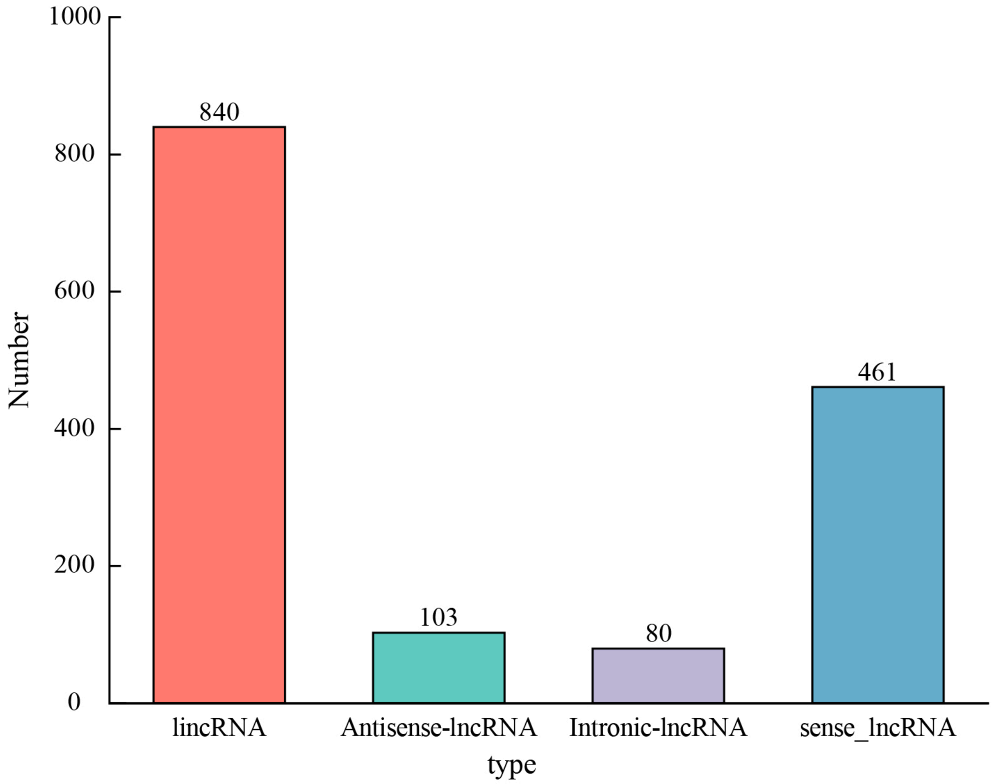

Figure S6. Predicted LncRNA Position Classification Map.

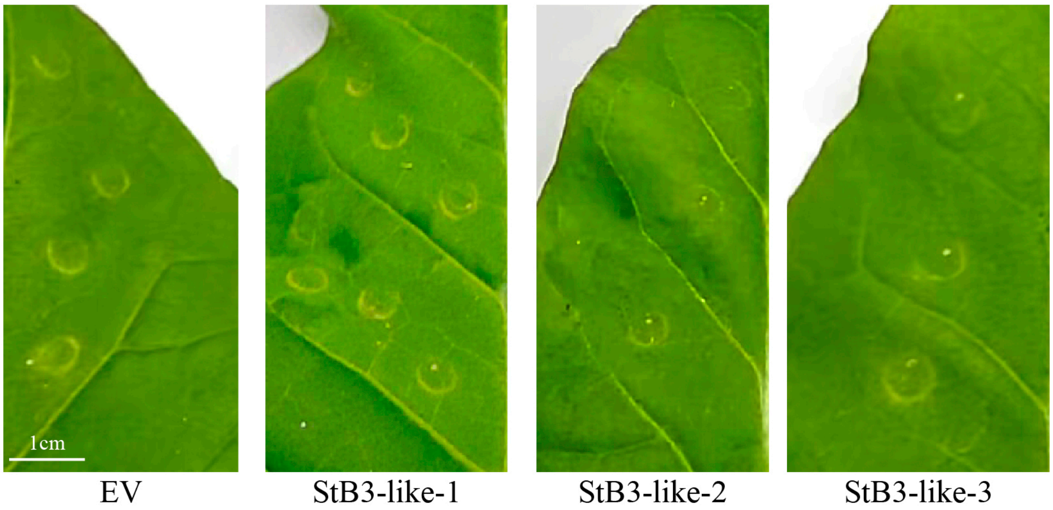

Figure S7. Phenotypes of StB3-like-1, StB3-like-2, and StB3-like-3 when injected individually into tobacco leaves.

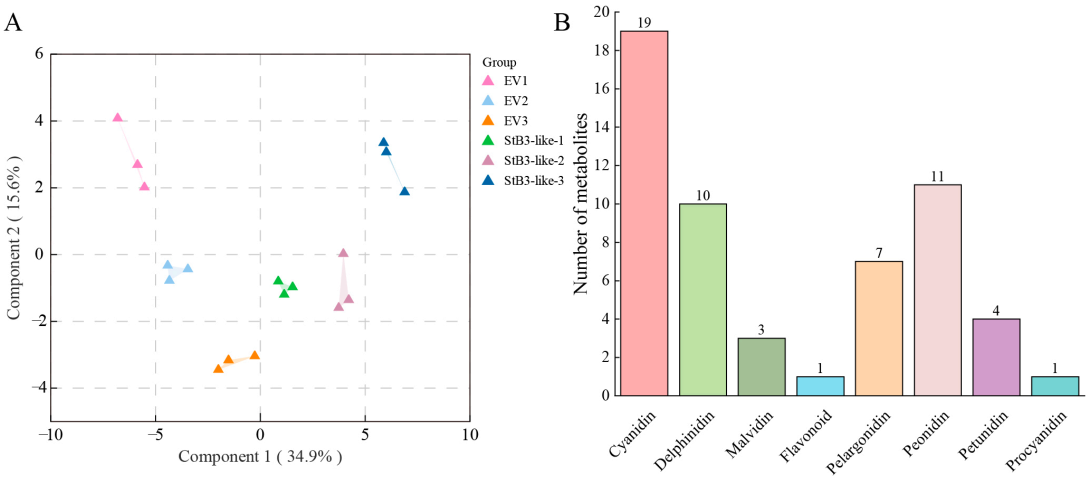

Figure S8. Phaniditin metabolomics analysis of leaves injected with different transcripts. A: OPLS-DA analysis across all samples. B: Classification of all phaniditins detected in EV, StB3-like-1, StB3-like-2, and StB3-like-3.
